# Endothelium‐Treg Communication Through Extracellular Vesicle Transfer Exacerbates Acute Respiratory Distress Syndrome

**DOI:** 10.1002/jev2.70235

**Published:** 2026-02-08

**Authors:** Xu Liu, Wei Huang, Xiwen Zhang, Shiming Li, Haofei Wang, Zongsheng Wu, Shuangfeng Zi, Lu Wang, Ling Liu, Yi Yang, Jianfeng Xie, Mingzhu Zheng, Jie Chao, Haibo Qiu

**Affiliations:** ^1^ Jiangsu Provincial Key Laboratory of Critical Care Medicine, Department of Critical Care Medicine, Zhongda Hospital, School of Medicine Southeast University Nanjing Jiangsu China; ^2^ Department of Respiratory Medicine Zhongda Hospital of Southeast University Nanjing Jiangsu China; ^3^ Department of Pathogenic Biology and Immunology, School of Medicine, Jiangsu Provincial Key Laboratory of Critical Care Medicine Southeast University Nanjing Jiangsu China; ^4^ Department of Physiology, School of Medicine Southeast University Nanjing China

**Keywords:** ALI, ARDS, regulatory T cells, pulmonary endothelium‐derived EVs, IL‐21, Med1

## Abstract

Decreased percentage of Foxp3^+^ regulatory T cells (Tregs) in the lungs results in overwhelming inflammation and delayed recovery of acute lung injury (ALI) caused by acute respiratory distress syndrome (ARDS). Extracellular vesicles (EVs) in the pulmonary microenvironment significantly affect the immune system, but their underlying effects on Tregs are unclear. Here, we demonstrate increased endothelial cell‐derived EVs (CD31^+^ EVs) in lipopolysaccharide (LPS)‐induced ALI models by single‐EV analysis. EVs from activated pulmonary endothelial cells (ECs) exhibit proinflammatory effects and suppress Treg induction. Exposure of Tregs to these EVs induces massive production of interleukin (IL)‐21, which has been proven to reduce Foxp3 expression. Mechanistically, we find that Med1 enriched in these EVs can directly bind to the promoter region of the IL‐21 gene thus activating IL‐21 transcription in Tregs. Moreover, we confirm that suppressing Med1 accumulation in EVs from activated pulmonary ECs can reverse Treg differentiation, and alleviate lung inflammation. Finally, we observe a significant increase of EVs carrying Med1 in the BALF of patients with ARDS. Taken together, this study identifies that EV‐mediated pulmonary endothelium‐Treg communication is crucial for Treg suppression in ARDS and may provide potential therapeutic targets for the treatment of this fatal clinical syndrome.

## Graphical abstract

Here, we reveal that pulmonary endothelium‐derived EVs aggravate lung inflammation via destabilizing Tregs during ALI/ARDS. Mechanistically, Tregs absorbs EV‐Med1, which promotes the secretion of IL‐21. This cytokine in turn induces hyper‐phosphorylation of STAT3 in Tregs thus induing loss of Foxp3, creating an hyperinflammatory ALI/ARDS microenvironment.

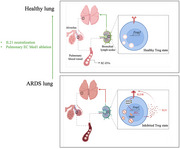

## 1 Introduction

Acute respiratory distress syndrome (ARDS), which is a life‐threatening form of acute lung injury (ALI) characterized by diffused pulmonary inflammation (Meyer et al. 2021; Matthay et al. 2024), is reportedly identified in approximately 10% of intensive care unit admissions, with a mortality around 40% (Gorman et al. 2022; Liu et al. 2018). Conventional CD4^+^ T cells are crucial effectors of adaptive immunity (Bonilla and Oettgen 2010), whereas naïve CD4^+^ T cell‐derived regulatory T cells (Tregs) suppress inflammation and promote tissue repair (Sakaguchi et al. 2010; Arpaia et al. 2015) and play a crucial role in ALI/ARDS (Halter et al. 2024). Patients with ARDS are demonstrated to have reduced Tregs in bronchoalveolar lavage fluid (BALF) (Halter et al. 2020), which correlated with relatively short survival and prolonged recovery (Norton et al. 2020). Impairments in the development, maintenance or functional capacity of Tregs are one of the major factors that drive ALI/ARDS progression, but the precise mechanisms remain largely unclear.

Extracellular vesicles (EVs) are nanoscale lipid‐bound particles that are secreted by various cells (van Niel et al. 2018) and act as critical mediators of inflammation by exerting pleiotropic effects on local or systemic immune responses (Buzas 2023). Our previous studies demonstrate that EVs from *ex vivo* human ARDS lungs induce tissue damage in healthy lungs (Liu et al. 2019, Zhang et al. 2021), and that EVs in an ALI pulmonary microenvironment decrease the repair efficiency of mesenchymal stem cells (MSCs) (Wu et al. 2024). Moreover, we observe that EVs are able to elicit strong innate immune responses during sepsis‐associated ALI (Wang et al. 2024). Accumulating evidences implicate the roles of the adaptive immune system in ALI/ARDS progression (Thompson et al. 2017; Unterman et al. 2022; D'Alessio et al. 2019). However, to date, there has been limited evidence to confirm the direct effects of EVs on adaptive Tregs during ALI/ARDS development. In addition, almost all pulmonary cells secrete EVs (Shah et al. 2018), but our understanding of the heterogeneous functions of EVs from different pulmonary cells in normal and ALI/ARDS pulmonary microenvironment remains largely unclear.

In this study, we reveal the importance of EVs secreted by pulmonary endothelial cells (ECs) in an ALI mouse model for ARDS by impairing Treg differentiation. In particular, EVs derived from pulmonary ECs are positively associated with the ALI severity and can specifically affect Tregs. Exposure of Tregs to EVs derived from activated pulmonary ECs significantly enhance interleukin (IL)‐21 production, which then stimulate STAT3 phosphorylation to downregulate Foxp3 expression. Furthermore, we identify that Med1 in these EVs is bound to the specific promoter regions of IL‐21 and facilitates the IL‐21 production in Tregs. In addition, inhibition of IL‐21 signalling in Tregs or Med1 expression in EVs derived from activated pulmonary ECs ameliorate murine ALI. Lastly, we find that pulmonary ECs are major sources of EVs carrying Med1, which are significantly elevated in the BALF of patients with ARDS. Taken together, our findings demonstrate the role of pulmonary EC‐Med1‐IL21‐Treg axis in ALI/ARDS.

## 2 Methods

### 2.1 Animals

Male C57BL/6 6–8‐week‐old mice were purchased from Hangzhou Ziyuan Laboratory Animal Technology Co., Ltd. (Zhejiang, China). *Il21* knockout mice were purchased from GenePharmatech Co., Ltd. (Jiangsu, China). *Tcrα* knockout mice and Rosa26‐LSL‐tdTomato were purchased from Cyagen Biosciences Inc. (Jiangsu, China). Foxp3^YFP/Cre^ mice were purchased from Jackson Laboratories (Bar Harbor, ME, USA).

For the ALI models, mice were anesthetized by pentobarbital sodium (40 mg/kg) and then intratracheally injected LPS (5 mg/kg, L2637, Sigma‐Aldrich, USA) dissolved in 100 µL sterile PBS through an endotracheal intubation. Following the procedures, mice were kept warm on a heating pad until completely alert and subsequently placed back in their cages before being sacrificed. After sacrifice, BALF, lung tissues, and BLNs were collected for further experiments. For survival studies, intratracheal LPS administration was used at a dose of 25 mg/kg body weight per mouse.

For the chimeric models, recipient mice were exposed to a total of 5‐Gy irradiation, then intravenously injected with BM cells (approximately 10^7^/per mouse) from donor mice. After 8 weeks, the chimera models were ready for further experiments.

For AAV‐mediated *in vivo* Med1 knockdown in pulmonary endothelium, mice were intravenously injected with shMed1‐AAV‐LungX or shNC‐AAV‐LungX (5 × 10^11^ vg/per mouse) (Obio Technology, Shanghai, China). After 4 weeks, mice were proceeded to further experiments.

All mice were bred and maintained under specific pathogen‐free (SPF) conditions in the Division of Laboratory Animal Center of Southeast University. All animal experimental protocols were approved by the Institutional Animal Care and Use Committee of Southeast University (No. 20220227005).

### 2.2 PBA

The PBA‐based single EV sequencing and analysis were performed, as previously described (Wu et al. 2019). Briefly, six mice were randomly allocated to the control or ALI group. The normal group (*n* = 3) acted as the control group, whereas the ALI groups were subjected to LPS intratracheally‐injection and sacrificed at 24 h (*n* = 3). The BALF from the same group were pooled and proceeded to sequentially centrifuge at 400 × *g* for 5 min to remove dead cells, 2000 × *g* for 20 min to remove debris and 13,000 × *g* for 30 min to remove large EVs. Then the supernatants were ultracentrifuged at 200,000 × *g* for 2 h at 4°C (A99846 equipped with a Type 45 Ti rotor, Beckman Coulter, USA). The isolated EVs pellets were first incubated with a total of 250 oligonucleotides labelled antibodies for protein probing. Then the EVs are captured in microtiter wells via immobilized coating of biotinylated cholera toxin subunit B (biotin‐CTB). 14593463 EVs were captured in control group and 12127119 EVs were captured in ALI group. When the Barcoding template was added, the oligonucleotides on the antibody hybridized to a specific sequence within the barcoding template. With DNA polymerase, the oligonucleotides on the antibody underwent extension reaction to obtain EV tag, which was the reverse complementary sequence of a designed part in barcoding template. DNA fragment library was constructed for high‐throughput sequencing. Extraction of EV tags and protein tags for sequencing reads were performed to generate the raw PBA data for further analysis. All experiments and data analysis were prepared and conducted by SecreTech, Inc., (Shenzhen, China) under the instructions of Vesicode AB (Solna, Sweden). The 250 oligonucleotides labelled antibodies used in this experiment were listed in Table S3.

### 2.3 FCM Bead Assays

Magnetic beads‐based EV surface protein detection was performed, as described elsewhere (Middleton et al. 2018). In brief, BALF samples were sequentially centrifuged at 400 × *g* for 5 min to remove dead cells, 2000 × *g* for 20 min to remove debris and 13,000 × *g* for 30 min to remove large EVs. Thereafter, the supernatant was mixed with EV capture beads (297‐79701, WAKO, Japan) for 1 h at room temperature. The EV‐binding beads were isolated by magnet and washed for three times using 1× washing buffer (with EV binding enhancer). Subsequently, the EV‐binding beads were resuspended and stained with fluorescent antibodies for another 1 h at room temperature and proceeded to analyze using a flow cytometer (LSRFortessa, BD Bioscience, USA).

The following antibodies were used to stain the surface proteins of mouse BALF‐EVs: BV421 Rat Anti‐Mouse CD9 (564235, BD Bioscience, USA); FITC Rat Anti‐Mouse CD63 (143919, BioLegend, USA); APC Rat Anti‐Mouse CD81 (551112, BD Bioscience, USA); and PE Rat Anti‐Mouse CD31 (553373, BD Bioscience, USA).

The following antibodies were used to stain the surface proteins of human BALF‐EVs: PE Mouse Anti‐Human CD9 (555372, BD Bioscience, USA); PE Mouse Anti‐Human CD63 (557305, BD Bioscience, USA); PE Mouse Anti‐Human CD81 (555676, BD Bioscience, USA); and PE Mouse Anti‐Human CD31 (566125, BD Bioscience, USA).

### 2.4 FCM Analysis

Tregs in BALF and BLNs and those polarized *in vitro* were stained and analyzed by FCM, as described previously with some modifications. Cells were blocked in PBS with Purified Rat Anti‐Mouse CD16/CD32 (553141, BD Bioscience, USA) for 10 min at room temperature and then stained with surface marker antibodies at 4°C for 30 min in a dark place. Thereafter, the cells were washed with PBS and proceeded to be fixed, permeabilized and stained with PE Rat Anti‐Mouse Foxp3 (12‐5773‐82, Invitrogen, USA) or eFluro450 Rat Anti‐Mouse IL‐21 (48‐7211‐82, Invitrogen, USA) using Transcription Factor Staining Buffer Kit (00‐5523‐00, Invitrogen, USA), according to the manufacturer's instructions. Subsequently, the cells were washed two times with 1× permeabilization buffer and analyzed using the LSRFortessa flow cytometer.

The following cell surface marker antibodies were used for staining of Tregs in mouse BALF and BLNs: Fixable Viability Stain 780 (565388, BD Bioscience, USA); BV510 Rat Anti‐Mouse CD45 (103137, BioLegend, USA); and BV786 Rat Anti‐Mouse CD4 (553047, BD Bioscience, USA).

The following cell surface marker antibodies were used to stain mouse Tregs that were polarized *in vitro*: BV421 Rat Anti‐Mouse CD4 (562891, BD Bioscience, USA); and APC Rat Anti‐Mouse CD25 (17‐0251‐82, Invitrogen, USA).

### 2.5 Cell Culture and EV Isolation

Mouse pulmonary microvascular ECs (MPMECs) were previously established (Liu et al. 2021) and maintained in DMEM‐F12 (C11330500BT, Gibco, China) supplemented with 5% (v/v) heat inactivated fetal bovine serum (FBS) (F8318, Sigma‐Aldrich, Australia) and 1% (v/v) penicillin‐streptomycin (P/S) solution (1514012, Gibco, USA). For generation of GFP‐CD63 MPMECs, cells were transfected with a GFP‐tagged CD63 gene using a lentivirus vector system (Abm Inc., Zhenjiang, China). For establishment of 3×Flag‐Med1 MPMECs, cells were transfected with a 3 × Flag‐tagged Med1 gene using a lentivirus vector system (GeneChem Inc., Shanghai, China). For generation of Med1‐knockdown MPMECs, cells were transfected with a shMed1 recombinant lentivirus (Abm Inc., Zhenjiang, China). To maintain gene expression or silencing, all cells that under lentiviral transduction were cultured in a medium containing puromycin (ST551, Beyotime, China) to maintain gene expression or silencing.

For collection of EVs, cells were cultured in DMEM‐F12 supplemented with EV‐depleted FBS w/wo LPS (1 µg/mL) challenging. After 24 h, supernatants were collected for EV isolation, as previously described. Briefly, the supernatants were centrifuged at 400 × *g* for 5 min to remove dead cells, 2000 × *g* for 20 min to remove debris, 13,000 × *g* for 30 min to remove large EVs. Then the freshly prepared supernatants were ultracentrifuged at 200,000 × *g* for 2 h at 4°C (A99846 equipped with a Type 45 Ti rotor, Beckman Coulter, USA). The pellets were resuspended in 60 mL 4°C sterile PBS and washed twice by ultracentrifugation at 200,000 × *g* for 1 h at 4°C. Then the supernatants were discarded and EVs were re‐suspended in 100 µL sterile PBS for further experiments.

### 2.6 EV Characterization

EV morphology was evaluated and imaged by transmission electron microscopy (TEM). Briefly, 10 µL freshly prepared EVs were fixed with 10 µL 4% paraformaldehyde solution for 30 min at room temperature. The mixture was then placed onto a TEM copper grid and stained with 2% uranyl acetate. The grid was washed thrice by PBS, air‐dried and proceeded to observe (supported by Wuhan Servicebio Technology Co., Ltd.).

The size range and particle concentrations of the EVs was evaluated by nanoparticle tracking analysis, based on our previous report.

Protein concentrations of EVs were determined using the bicinchoninic acid assay (BCA) method, according to the manufacturer's instructions (KGB2101, KeyGEN, China).

### 2.7 EV Permeabilization and Proteinase K Digestion

A total of 20 µL 0.5% Triton X‐100 (Beyotime, China) in PBS was added into 80 µL EV sample and incubated for 10 min on the ice to permeabilize the EVs. The mixture was immediately diluted with 60 mL 4°C sterile PBS and proceeded to ultracentrifuge at 200,000 × *g* for 1 h at 4°C to washed away Triton‐X 100. The EVs pellet was resuspended in 100 µL sterile PBS. Proteinase K (25530049, Invitrogen, USA) was added to digest EV proteins at a final concentration of 100 µg/mL at 56°C for 6 min. The Proteinase K was washed away with 60 mL 4°C sterile PBS by ultracentrifugation at 200,000 × *g* for 1 h at 4°C. The supernatant was discarded and the EVs pellet were resuspended in 100 µL sterile PBS for further experiments.

### 2.8 Treg Polarization *In Vitro*

Naïve CD4^+^ T cells were isolated and differentiated into Tregs *in vitro*, as previously described with some modifications. In brief, naïve CD4^+^ T cells were negatively separated from mice spleen samples, according to the manufacturer's instructions (19765, STEMCELL, USA). For *in vitro* polarization, 24‐well culture plates were precoated with Anti‐Mouse CD3 (2 µg/mL, 1 mL per well, 05112‐25, Biogems, USA) overnight at 4°C and washed three times with PBS prior to cell seeding. The isolated naïve CD4^+^ T cells were seeded at a density of 4 × 10^5^ per well and cultured in 1 mL RPMI‐1640 (36750, STEMCELL, USA) supplemented with 10% (v/v) FBS (F8318, Sigma‐Aldrich, Australia); 1% (v/v) P/S (1514012, Gibco, USA); 1% (v/v) Treg differentiation supplement (10957, STEMCELL, USA); 1% (v/v) GlutaMAX (35050061, Gibco, USA); 1 mM sodium pyruvate (11360070, Gibco, USA); 50 µM 2‐mercaptoethanol (21985023, Gibco, USA); and Anti‐Mouse CD28 (1 µg/mL, 05112‐25, Biogems, USA), with 10 µg EC‐EVs or LPS‐EC‐EVs simultaneously. Half of the culture medium was changed at Day 2, and cells were harvested at Day 5 for further experiments.

### 2.9 Coomassie Blue Staining, Ponceau S Staining and Western Blotting

The proteins in cells and EVs were extracted using NETN or RIPA lysis buffer supplemented with phenylmethyl sulfonyl fluoride and phosphatase inhibitors, depending on the different target molecules. Proteins were quantified using the KeyGEN BCA Protein Assay Kit, denatured and then separated on SDS‐polyacrylamide gels.

For Coomassie Blue staining, the SDS‐polyacrylamide gels were washed three times in ultrapure water and stained using Coomassie Blue solution (P0003S, Beyotime, China) for 20 min at room temperature. The SDS‐polyacrylamide gels were washed with ultrapure water and visualized using a gel imaging system (BIO‐OI, China).

For Ponceau S staining, the proteins were transferred onto PVDF membranes and incubated with Ponceau S solution (P0022, Beyotime, China) for 10 min at room temperature. PVDF membranes were washed with distilled water under manufacturer's instructions and proceeded to visualize.

For immunoblotting, PVDF membranes were blocked in Fast Blocking Buffer (G2052, Servicebio, China) for 10 min at room temperature and proceeded to incubate with primary antibodies at 4°C overnight. On the second day, the PVDF membranes were washed with 1× PBST solution and proceeded to incubate with secondary antibodies for 1 h at room temperature. PVDF membranes were washed with 1× PBST solution and analyzed using a Chemi4800mini imaging system (Bioshine, China). The following antibodies were used for the immunoblotting experiments: phosphor‐smad2 rabbit mAb (18338, CST, USA); smad2 rabbit mAb (5339, CST, USA); phosphor‐smad3 rabbit mAb (9520, CST, USA); smad3 rabbit mAb (9523, CST, USA); phosphor‐STAT5 rabbit mAb (25656, CST, USA); STAT5 rabbit mAb (94205, CST, USA); phosphor‐STAT3 rabbit mAb (9145, CST, USA); STAT3 rabbit mAb (9139, CST, USA); Foxp3 rabbit mAb (ab215206, Abcam, USA); Med1 rabbit mAb (A700‐037‐T, Bethyl Laboratories, USA); Flag tag rabbit mAb (14793, CST, USA); α‐tubulin rabbit pAb (11224‐1‐AP, proteintech, China); histone‐H3 rabbit pAb (17168‐1‐AP, proteintech, China); GM130 rabbit mAb (70767, CST, USA); Alix rabbit mAb (ab235377, Abcam, USA); CD63 rabbit pAb (ab315108, Abcam, USA); CD9 rabbit mAb (ab307085, Abcam, USA); β‐actin rabbit pAb (HPR conjugated) (ZF2001, ZFdows Bio, China) and HPR‐linked anti‐rabbit Ab (7074, CST, USA).

### 2.10 Histopathology, Immunohistochemistry and Immunofluorescence

Freshly isolated tissues were fixed in 4% paraformaldehyde for more than 96 h at room temperature and then processed into paraffin‐embedding.

For HE staining, the paraffin‐embedded lung tissues were sliced into 4 µm thick sections and stained with the haematoxylin and eosin dye directly.

For IHC, paraffin‐embedded lung sections were dewaxed, retrieved, permeabilized and blocked before overnight incubation with Ly6G rabbit mAb (ab238132, Abcam, USA) or Neutrophil Elastase rabbit mAb (A8953, Abclonal, China). The next day, the sections were incubated with secondary antibodies.

For immunofluorescence, sections were permeabilized and blocked before overnight incubation with MPO rabbit mAb (ab208670, Abcam, USA) or CD4 rabbit pAb (ab288725, Abcam, USA). The next day, the sections were incubated with fluorescent dry‐conjugated secondary antibodies.

### 2.11 SEM and IEM

For SEM, Tregs exposed to EC‐EVs or LPS‐EC‐EVs were harvested and then fixed in 2% paraformaldehyde. The samples were washed and diluted in distilled water. The sample were placed on silicon chips before being sputtered on 2–5 nm gold‐palladium alloy for imaging.

For IEM, EVs were dried and permeabilized on nickel grids, then incubated with Med1 rabbit mAb (A700‐037‐T, Bethyl Laboratories, USA) at 4°C. After 6 h, the grids were washed with PBS and incubated with secondary antibodies conjugated to gold particles for 2 h, followed by staining with 2% phosphor‐tungstic acid solution. The SEM and TEM were both supported by Wuhan Servicebio Technology Co., Ltd.

### 2.12 Cell Proliferation Assays

Isolated naïve CD4^+^ T cells were stained with CFSE (KGA9503, KeyGEN, China), according to the manufacturer's instructions, and then polarized into Tregs for 5 days with either EC‐EVs or LPS‐EC‐EVs. Cells were harvested at Day 5 and quantified through the LSRFortessa flow cytometer.

### 2.13 Apoptosis Assay

Isolated naïve CD4^+^ T cells were polarized into Tregs for 5 days with either EC‐EVs or LPS‐EC‐EVs. Cells were harvested at Day 5 and stained with Annexin V‐PI (C1062S, Beyotime, China) to evaluate the percentage of apoptotic cells using Novocyte flow cytometer. The staining procedures were strictly performed, according to the manufacturer's instructions.

### 2.14 ELISA Assay

To measure cytokine production, naïve CD4^+^ T cells were polarized under various Th differentiation conditions with either EC‐EVs or LPS‐EC‐EVs in ImmunoCult‐XF T cell expansion medium (10981, STEMCELL, USA). Supernatants were collected at Day 5, and the concentration of TGF‐β1 (DB100C, R&D, USA), KGF (ELK3081, ELK Biotech, China), IFN‐γ (E‐EL‐M0048, Elabscience, China), IL‐4 (E‐EL‐M0043, Elabscience, China), IL‐17A (E‐EL‐M0047, Elabscience, China) and IL‐21 (EK0797, BOSTER, China) were determined by ELISA assays, according to the respective manufacturers’ instructions.

### 2.15 RT‐qPCR

The SPARKeasy kit (ACS201, SparkJade, China) was used to extract total mRNA from tissues or cells, following the manufacturer's instructions. The mRNA was transformed into cDNA using HiScript III RT SuperMix (R323, Vazyme, China), and then subjected to real‐time polymerase chain reaction using ChamQ SYBR qPCR Master Mix (Q341‐02, Vazyme, China). The expression of genes was normalized to *β‐actin*. The primer sequences of all genes were listed in Table S4.

### 2.16 mRNA Sequencing

Isolated naïve CD4^+^ T cells were polarized into Tregs for 5 days with either EC‐EVs or LPS‐EC‐EVs. Cells were harvested at Day 5 and total RNA was extracted using a Total RNA Purification Kit (LC Science, USA). A mRNA library was constructed using the TruSeq Standed mRNA LT Sample Prep Kit (Illumina, USA) according to the manufacturer's instructions. The libraries were sequenced on an Illumina HisSeq X Ten platform by OEbiotech, Co., Ltd (China). Differential expression analysis was performed using the DESeq R package.

The raw mRNA‐seq data have been uploaded to the National Center for Biotechnology Information (NCBI) project PRJNA1258061. All data are publicly available since the date of publication.

### 2.17 IPA

The upregulated genes in the Tregs exposed to LPS‐EC‐EVs were imported into IPA software (www.ingenuity.com/, Qiagen, USA) and subjected to diverse analyses or predictions, such as graphical summaries, mechanistic networks and upregulated regulators, based on precise algorithms.

### 2.18 Confocal Microscopy

Treg pellets were resuspended and fixed in 4% paraformaldehyde for 10 min at room temperature. Thereafter, cell suspensions were placed onto a Poly‐l‐Lysine‐charged coverslip and air‐dried at room temperature. After permeabilizing and blocking, the cells were incubated with primary antibodies overnight at 4°C. The second day, cells were washed with PBS and incubated with fluorescent dye‐conjugated secondary antibodies for 1 h at room temperature. The coverslips were washed with PBS for 5 times and mounted on Fluoroshield with DAPI (F6057, Sigma‐Aldrich, USA) before imaging.

### 2.19 Nuclear and Cytoplasmic Protein Extraction

Isolated naïve CD4^+^ T cells were polarized into Tregs for 5 days with Flag‐Med1 EVs. Cells were collected at Day 5 and extracted nuclear/cytoplasmic proteins using an extraction kit (KTP3001, Abbkine, China) according to the manufacturer's instructions. The separated nuclear and cytoplasmic proteins were quantified using KeyGEN BCA Protein Assay Kit and denatured by boiling with loading buffer (NP0007, Invitrogen, USA).

### 2.20 ChIP‐qPCR

ChIP analysis was performed using a commercial kit (53035, Active Motif, USA). Briefly, isolated naïve CD4^+^ T cells were polarized into Tregs for 5 days with Flag‐Med1 EVs. Cells were fixed at Day 5 with 1% paraformaldehyde for 10 min at room temperature and proceeded to chromatin preparation according to the manufacturer's instructions. Flag rabbit antibody (14793, Cell Signaling, USA) and IgG rabbit antibody (2729, Cell Signaling, USA) were used to perform the pulldown. The immunoprecipitated DNA was directly used for qPCR analysis. The primers used in this experiment were as follows:

*Il21* site1
F‐ ACCCCCAAATTCTGTCCGAT
R‐GGAGACAAGGTCCAATAGGAGTAG;
*Il21* site2
F‐ TTTCTGACTGTATAAGCAAGCG
R‐GAAAAGGCACAAGAAGCATATAG;
*Il21* site3
F‐GCACCGTCAGATTTCAGAGAAGT
R‐TCAAAAAGCATAGTCATCACCCCAT;
*Gapdh*
F‐ CCCTTGAGCTAGGACTGGA
R‐ TTTATAGGAACCCGGATGG.

### 2.21 Agarose Gel Electrophoresis

The ChIP‐qPCR products mixed with loading buffer were added to 1% agarose gels containing ethidium bromide. Electrophoresis was stopped when the leading dye reached the end of the gel and then the image was acquired using a gel imaging system (BIO‐OI, China).

### 2.22 Dual‐Luciferase Assays

IL‐21 or IL‐21 mutation was amplified, inserted into dual‐luciferase reporter vectors and co‐transfected into HEK293T cells with Med1 or blank plasmids using a DNA/RNA transfection reagent (AD600100, Zeta‐life, USA). After 48 h, the luciferase activities were measured by a dual‐luciferase reporter assay system (E2940, Promega, USA) according to the manufacturer's instructions. The results were normalized to Renilla luciferase activity. All plasmids were provided by Corues Biotechnology (China).

### 2.23 Patient Enrolment

A total of nine patients who met the following criteria were included in this study: (1) adult older than 18 years; (2) diagnosis as ARDS according to the Berlin criteria no more than 72 h of admission; (3) patients in which an artificial airway was established. The exclusion criteria were as follows: (1) patients with chronic pulmonary diseases; (2) patients with autoimmune diseases; (3) patients who were pregnant; (4) contraindications to fibro‐bronchoscope examinations; (5) an expected survival of less than 24 h.

A cohort of five patients who underwent uvulo‐palato‐pharyngoplasty with incubation and mechanical ventilation, but without any pulmonary diseases, were enrolled as controls.

The use of the samples from patients at Zhongda Hospital Affiliated to Southeast University was approved by Research Ethics Board of Zhongda Hospital (Southeast University, Nanjing, China, 2021ZDSYLL215‐P01).

### 2.24 Human Samples

The BALF samples were collected within 24 h of initiation of incubation and mechanical ventilation. A total of 40 mL normal saline was used to lavage in the right middle and left upper lobes, and more than 40% of the fluid was collected. The samples were proceeded to centrifuged at 400 × *g* for 5 min to remove dead cells, 2000 × *g* for 20 min to remove debris, 13,000 × *g* for 30 min to remove large EVs, immediately. A total of 10 mL of the supernatants (the remainder were ready for magnetic beads‐based EV surface protein detection) were proceeded to isolated BALF‐EVs via ultracentrifugation at 200,000 × *g* for 2 h at 4°C and the EV pellets were used for western blotting.

### 2.25 Statistical Analysis

The mean ± SD of the data was shown in the bar graphs. Two datasets were compared using a Student's *t*‐test or unpaired *t*‐test. Multiple comparisons were conducted using analysis of variance, followed by either Dunnett's test or Bonferroni correction. Prism 7.0 (GraphPad Software, USA) was used for statistical analyses. A *p* value of <0.05 was deemed statistically significant. For the IPA analyses, a z‐score >2 or <–2 was recognized as statistically significant.

## 3 Results

### 3.1 EVs Derived From Pulmonary Endothelium Are Inversely Related With BALF and BLN Treg Proportion in ALI Mice

**FIGURE 1:**
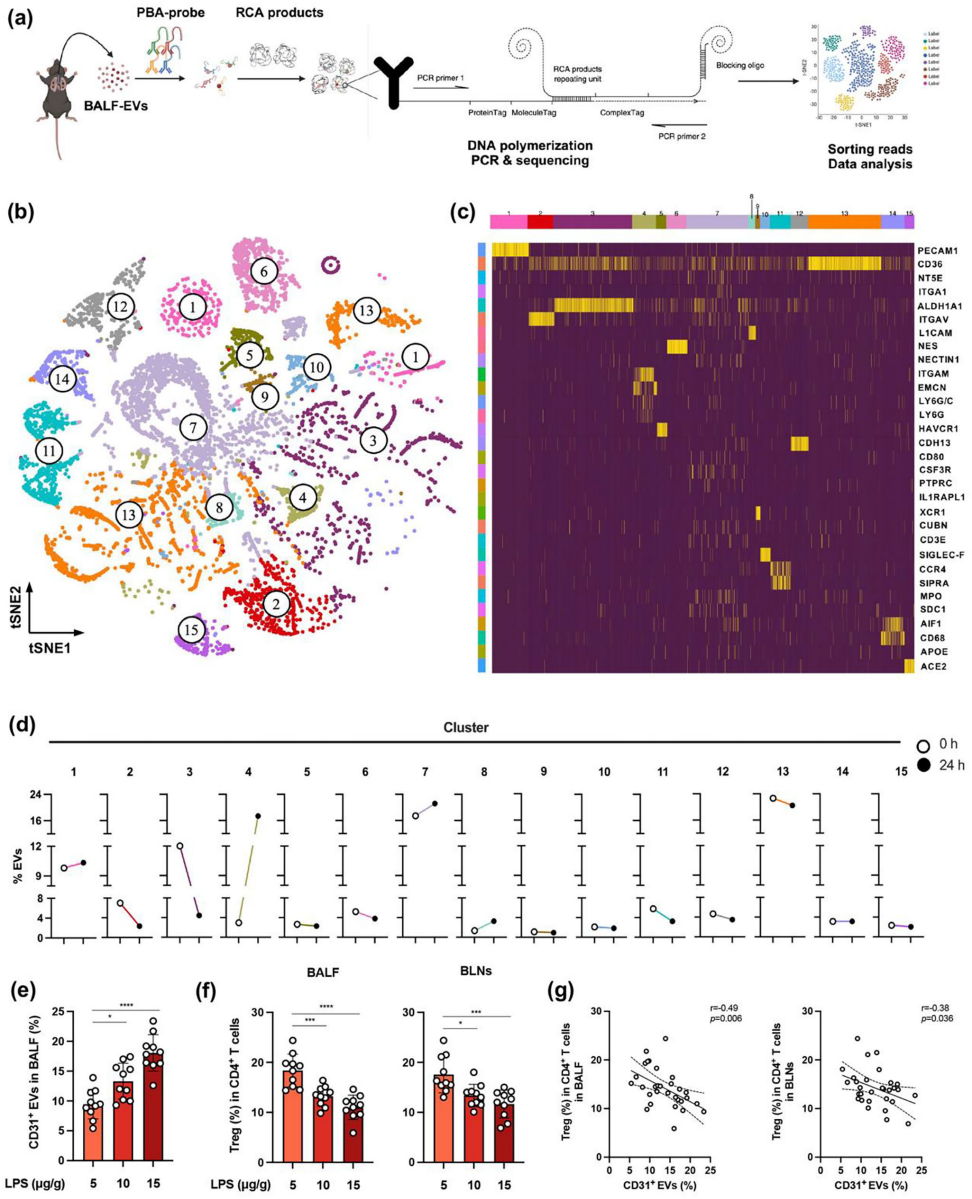
CD31^+^ EVs negatively correlate with BALF and BLN Treg proportion in LPS‐IT mice. (a) The working flowchart of PBA. For each experimental condition, the BALF EVs were pooled from three mice in each group (created using Biorender.com). (b) t‐SNA plot showed 15 EV subgroups from all analyzed samples. (c) Heatmap showed proteomic biomarker of each cluster. (d) Comparison of each cluster between the control group and LPS‐IT group. (e) Quantification of CD31^+^ EV percentage in the BALF of mice after administration of LPS intratracheally (*n* = 10 each group). (f) Tregs frequency in the BALF (left) and BLNs (right) of mice with different ALI severities (*n* = 10 each group). (g) Correlations of CD31^+^ EVs with BALF Tregs (left) and BLN Tregs (right). *p < 0.05, ***p < 0.005, ****p < 0.001. See also Figures S1–S5.

To characterize the production and changes in pulmonary EVs during ALI/ARDS, we performed proximity barcoding assay (PBA) to profile single EV that was isolated from the BALF samples of control mice and ALI mice induced by lipopolysaccharide intratracheally administration (LPS‐IT) (Figure 1a). An unsupervised machine learning algorithm named FlowSOM generated 15 major EV clusters (Figure 1b). The percentile and representative surface proteins of each subcluster were presented (Figure 1c and Figure S1). Eleven clusters were characterized with subpopulation‐specific protein, including cluster 1 (dominantly expressed PECAM1/CD31), cluster 2 (dominantly expressed ITGAV), cluster 3 (dominantly expressed ALDH1A1), cluster 5 (dominantly expressed HAVCR1/TIM‐1), cluster 6 (dominantly expressed Nestin), cluster 8 (dominantly expressed L1CAM), cluster 9 (dominantly expressed XCR1), cluster 10 (dominantly expressed SIGLEC‐F), cluster 12 (dominantly expressed CDH13), cluster 13 (dominantly expressed CD36) and cluster 15 (dominantly expressed ACE2). However, clusters 4, 7, 11 and 14 were lack of predominant protein biomarker, because the frequencies of featured proteins in these clusters were all lower than the threshold 0.7. Among 15 clusters, the percentage of EVs was higher in the LPS‐IT group than in the control group in cluster 1(9.81% vs. 10.34%), cluster 4 (2.94% vs. 17.36%), cluster 7 (17.43% vs. 21.17%), cluster 8 (1.35% vs. 3.25%) and cluster 14 (3.11% vs. 3.12%), but was lower in the LPS‐IT group than in the control group in the remaining clusters (Figure 1d). These results indicated dynamic and altered proteomic characteristics of EVs during ALI/ARDS development.

**FIGURE 2:**
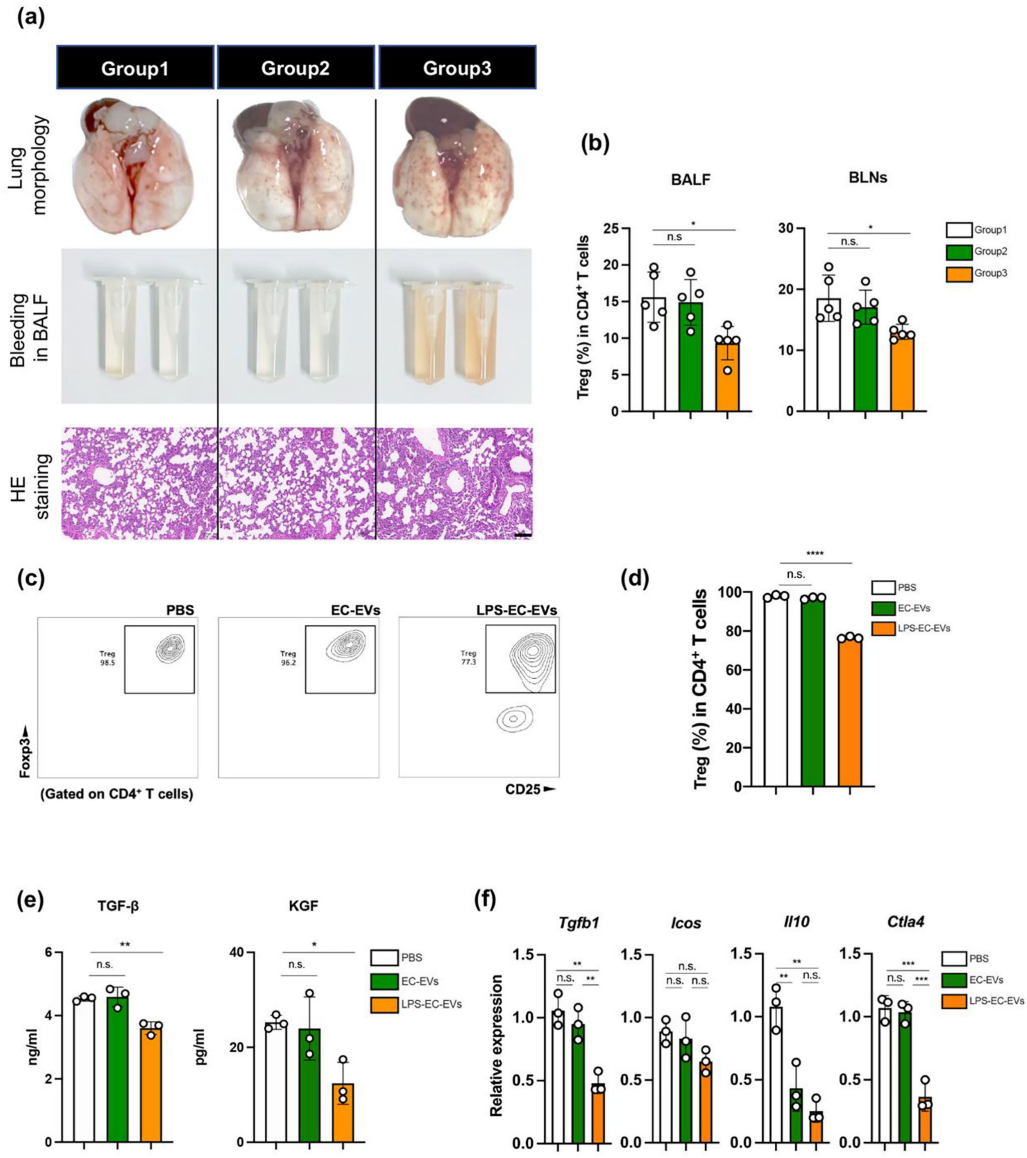
LPS‐EC‐EVs inhibit Treg polarization. (a) LPS‐EC‐EVs augment the lung injury of LPS‐IT mice. (Upper) Gross pathologic findings; (middle) bleeding in BALF; (lower) histological examination; scale bar, 100 µm. (b) Tregs proportion in BALF (left) and BLNs (right) of mice in different group (*n* = 5 each group). (c) FCM and (d) quantification of Foxp3^+^ cells *in vitro* Treg analysis (*n* = 3). (e) TGF‐β1 and KGF concentration in culture medium from each group (*n* = 3). (f) RT‐qPCR analysis of signature genes of Tregs in each group (*n* = 3). *p < 0.05, **p < 0.01, ***p < 0.005, ****p < 0.001. See also Figures S6–S11.

Among the elevated BALF‐EV subpopulations in LPS‐IT mice, we focused on cluster 1, which occupied nearly 10% in total EVs. EVs frequently carry identical markers as their parent cells. The EVs in cluster 1 expressed high levels of CD31, which is a typical marker used to identify the endothelial cell origin of EVs (Thomashow et al. 2013; Amabile et al. 2014; Parker et al. 2014). Although clusters 4 and 7 had higher proporation, we failed to identify their cell origins because they exhibited no obvious surface markers for assignment. Moreover, previous studies demonstrate that the pulmonary endothelium can secrete EVs to orchestrate the lung microenvironment (Letsiou et al. 2015; Feng et al. 2021). Therefore, we defined and chose cluster 1, endothelial‐derived EVs (Figure S2a,b), for further exploration. The EV capture bead‐based flow cytometry (FCM) demonstrated that the proportion of these EVs in BALF (Figure 2c,d) increased with the severity of lung injury (Figure 1e and Figure S2e,f). These results suggested that the progression of ALI/ARDS was associated with increased secretion of EVs from pulmonary ECs.

We then explored the function of EVs derived from activated pulmonary ECs in ALI/ARDS. To simplify our study, we used LPS to activate a mouse pulmonary endothelial cell line established by our laboratory before (Liu et al. 2021) *in vitro*, and collected EVs from cell culture supernatants (LPS‐EC‐EVs). We first characterized the size, morphology, and protein marker expressions of these LPS‐EC‐EVs (Figure S3a–c). We next investigated the role of LPS‐EC‐EVs *in vivo*. Compared with mice injected with LPS alone, C57BL/6 mice that received LPS plus LPS‐EC‐EVs had more severe lung inflammation, as evidenced by increased mRNA levels of interleukin (IL)‐6, IL‐1β and tumour necrosis factor (TNF)‐α (Figure S3d). Moreover, pulmonary tissue damage and myeloperoxidase‐positive (MPO^+^) neutrophilic infiltration were amplified by additional administration of LPS‐EC‐EVs (Figure S3e,f). Collectively, these data indicated that LPS‐EC‐EVs promoted lung inflammation *in vivo*.

To further verify the effects of LPS‐EC‐EVs on pulmonary Tregs, we first examined the proportion of Tregs in BALF and bronchial lymph nodes (BLNs) from mice with different ALI severities (Figure S4). The percentage of Tregs in CD4^+^ T cells in BALF and BLNs decreased with increasing ALI severity (Figure 1f) which were consistent with the previous clinical observations. Further bivariate analysis showed the percentage of BALF CD31^+^ EVs negatively correlated with the Treg proportions in both BALF and BLNs (Figure 1g). Considering that Tregs mainly differentiate within lymphoid tissues (Miragaia et al. 2019), we then transfected the mouse pulmonary endothelial cell line with a GFP‐tagged CD63 gene using lentivirus (Figure S5a) and traced the distribution of GFP‐labelled LPS‐EC‐EVs *in vivo* (Figure S5b). ALI mice were injected intravenously with GFP‐labelled LPS‐EC‐EVs (100 µg per mouse) or sterile phosphate buffered saline (PBS) as control. The signals of GFP‐labelled LPS‐EC‐EVs were detected in the spleen samples and BLNs at 24 h after injection (Figure S5c,d), and immunofluorescence staining showed the colocalization of GFP‐labelled LPS‐EC‐EVs and CD4^+^ T cells in BLNs (Figure S5e). Taken overall, we deliberated that Treg generation might be affected by interaction with LPS‐EC‐EVs.

### 3.2 LPS‐EC‐EVs Specifically Restrain Treg Differentiation

To elucidate the influence of LPS‐EC‐EVs on Treg generation, we first pre‐challenged C57BL/6 mice with 100 µg of EVs from PBS‐treated pulmonary ECs (EC‐EVs) or LPS‐challenged pulmonary ECs (LPS‐EC‐EVs) twice, with the same amount of sterile PBS as a control. Then these mice were injected LPS intratracheally to induce ALI (Figure S6a). Compared with ALI mice pre‐treated with PBS or EC‐EVs, ALI mice pre‐treated with LPS‐EC‐EVs showed more severe lung damage (Figure 2a), demonstrating exacerbated pulmonary inflammation. Further, we quantified Treg populations in both BALF and BLNs, revealing a significant reduction in their proportions following pre‐challenged with LPS‐EC‐EVs (Figure 2b).

Next, we cocultured splenic naïve CD4^+^ T cells with EC‐EVs or LPS‐EC‐EVs (10 µg/mL culture medium) under Treg polarization condition *in vitro* for 5 days, with an equal amount of sterile PBS as a control (Figure S6b). After 24 h, EC‐EVs or LPS‐EC‐EVs were observed on the cell surface through scanning electronic microscopy (SEM), demonstrating the interaction of cells with EVs (Figure S7a). The survival of cells stimulated by anti‐CD3/anti‐CD28 were not affected by EC‐EVs or LPS‐EC‐EVs (Figure S7b). Neither EC‐EVs nor LPS‐EC‐EVs affected Treg proliferation (Figure S7c) and apoptosis (Figure S7d).

Subsequently, we evaluated Treg polarization efficiency using FCM. As expected, the percentage of Tregs was drastically decreased after 5 days of coculture with LPS‐EC‐EVs but not with EC‐EVs (Figure 2c,d). The degree of cytokine production (Figure 2e) and signature gene expression analyses (Figure 2f) were consistent with these results. Previous studies demonstrate that Tregs promote lung tissue repair through CD73 (Ehrentraut et al. 2013) and CD103 (Mock et al. 2014) expression. Therefore, we evaluated their expressions on Tregs. However, LPS‐EC‐EVs did not affect their expressions on Tregs (Figure S8a,b). We also evaluated the polarization of other T helper cells exposed to LPS‐EC‐EVs. Notably, LPS‐EC‐EVs did not affect Th2 and Th17 differentiation and only slightly affected Th1 differentiation (Figure S9a–c).

To further investigate whether LPS‐EC‐EVs impaired Treg stability, we employed a lineage‐tracing approach by crossing Foxp3^YFP/Cre^ mice with Rosa26‐LSL‐tdTomato mice (Figure S10a). Splenic naïve CD4^+^ T cells isolated from these mice were polarized into Tregs in the presence or absence of LPS‐EC‐EVs. The FCM results revealed that LPS‐EC‐EVs exposure significantly reduced the proportion of stable Tregs (YFP^+^ tdTomato^+^) while concomitantly increasing exTregs (YFP^−^ tdTomato^+^) (Figure S10b,c). These findings demonstrated that LPS‐EC‐EVs potently destabilized Tregs and suppress Foxp3 expression.

To exclude potential confounding effects of LPS contamination on Treg suppression, we performed multiple control experiments. First, we measured the endotoxin levels in LPS‐EC‐EVs and confirmed minimal residual endotoxin contamination (Table S1). Next, we generated control EVs (EC‐EVs(L)) by treating pulmonary ECs with PBS followed by exogenous LPS supplementation (1 µg/mL) prior to EV isolation (Figure S11a). In parallel Treg polarization assays, neither EC‐EVs (L) (10 µg per mL culture medium) nor direct LPS (100 ng/mL) treatment affected Treg polarization compared to PBS controls *in vitro* (Figure S11b), which was consistent with previous studies (Reynolds et al. 2012; Ding et al. 2020). Taken together, these comprehensive results confirmed that the observed Treg inhibition was specifically attributable to LPS‐EC‐EVs rather than free LPS contamination.

### 3.3 TGF‐β and IL‐2 Signalling Pathways Are Intact in EV‐Primed Tregs

T cell receptor (TCR) signalling plus transforming growth factor‐β (TGF‐β) signalling and IL‐2 signalling are crucial for Treg generation and expansion (Chinen et al. 2016; Konkel et al. 2017; Schmidt et al. 2015). Therefore, we next investigated the effects of LPS‐EC‐EVs on these intracellular signalling pathways in Tregs. There were no significant differences in the expressions of TGF‐β receptors I and II (TGF‐β RI, TGF‐β RII) between the Tregs treated with EC‐EVs and those treated with LPS‐EC‐EVs (Figure S12a,b). Moreover, IL‐2 receptor (IL‐2R) and CD69 expression were not affected by LPS‐EC‐EVs (Figure S12c,d). In the TGF‐β signalling pathway, smad2 and smad3 are phosphorylated by TGF‐β receptor activation, then translocate into the nucleus to modulate gene transcription. As shown in Figure S12e,f, phosphorylation of smad2 and smad3 in Tregs treated with LPS‐EC‐EVs was not obviously affected. IL‐2 induces phosphorylation of STAT5, which then translocates into the nucleus to support Foxp3 expressing. However, we also did not find inhibitory effects of LPS‐EC‐EVs on STAT5 phosphorylation (Figure S12g). Our results excluded the possibilities that suppression of Tregs by LPS‐EC‐EVs was due to impairments in TGF‐β, IL‐2 and TCR signalling cascades.

### 3.4 Tregs Primed With LPS‐EC‐EVs Produce Massive IL‐21

**FIGURE 3:**
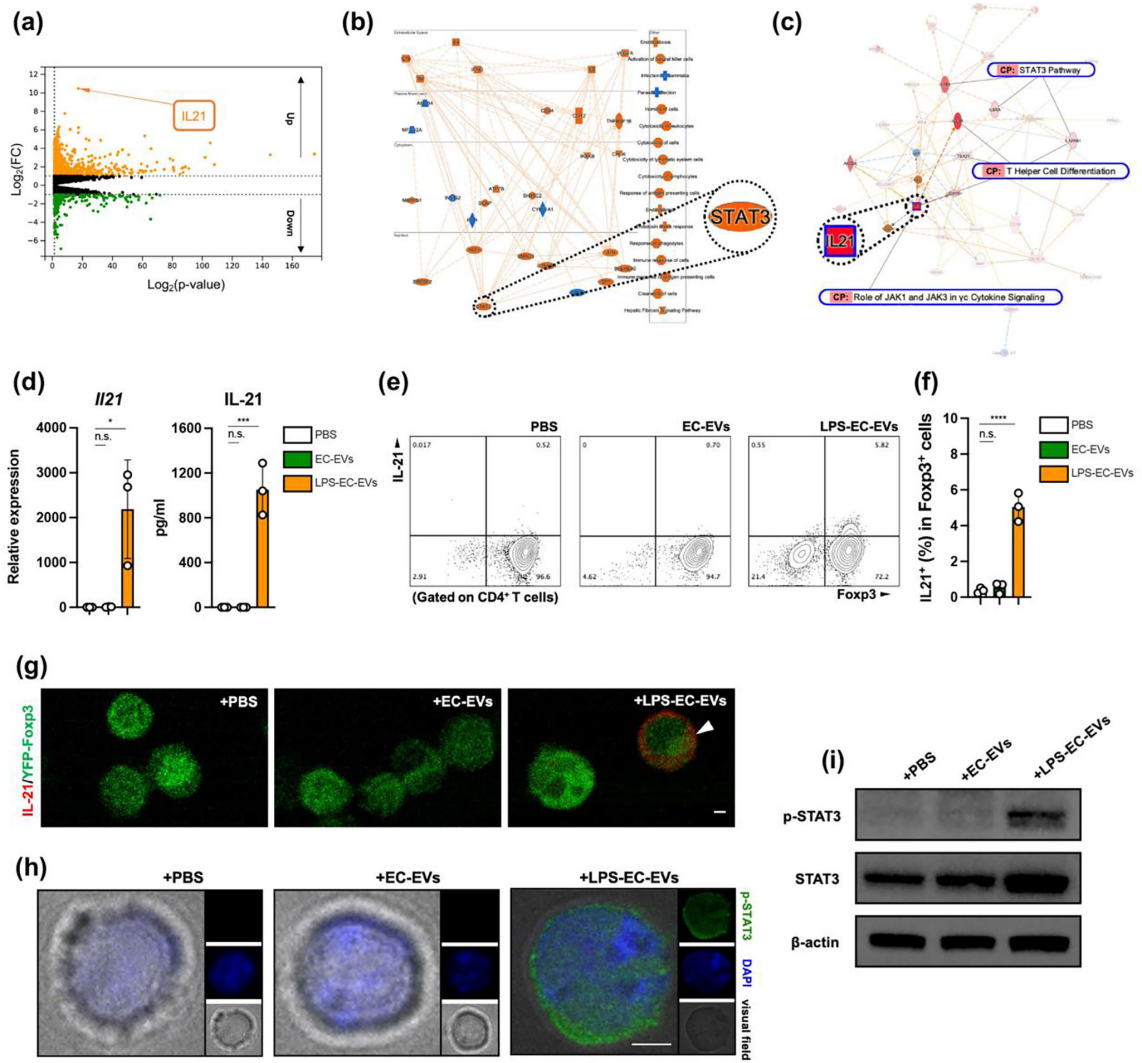
LPS‐EC‐EVs initiate IL‐21 production in Tregs. (a) Volcano plot of RNA‐seq expression in Tregs exposed to EC‐EVs or LPS‐EC‐EVs for 5 days. Orange dots indicated upregulated genes (fold change > 2, *p* value < 0.05), whereas green dots represented downregulated ones (fold change < 0.5, *p* value < 0.05) in Tregs with LPS‐EC‐EVs. (b) Graphic overview of the upregulated genes by IPA. (c) The IL‐21 centered molecular network generated by IPA. (d) RT‐qPCR combined ELISA analyses of IL‐21 expressions in Tregs exposed to either PBS or different EVs for 5 days (*n* = 3). (e) FCM and (f) quantification of IL‐21 staining in Tregs treated with either PBS or different EVs (*n* = 3). (g) Representative images of immunofluorescence staining of IL‐21 expression in Tregs *in vitro*. Naïve CD4^+^ T cells from Foxp3^YFP/Cre^ were polarized into Tregs exposed to either PBS or different EVs; Foxp3 (green), IL‐21 (red), Scale bar, 3 µm. (h) Representative confocal views and (i) Immunoblotting showing STAT3 activation exposed to LPS‐EC‐EVs (*n* = 3). In (h), Scale bar, 4 µm. *p < 0.05, ***p < 0.005, ****p < 0.001. See also Figure S13.

Considering that suppression of Tregs by LPS‐EC‐EVs was independent of the aforementioned cytokine signalling pathways, we performed global transcriptome analysis to further investigate the mechanism. Compared with Tregs treated with EC‐EVs, Tregs treated with LPS‐EC‐EVs had 709 genes that were significantly upregulated, and 209 genes that were obviously downregulated. Consistent with previous results, the expression of Treg signature genes, including *Foxp3*, were reduced in the LPS‐EC‐EVs group (Figure S13). Notably, of all the upregulated genes, only *Il21* was highly expressed over 1000 times (Figure 3a). IL‐21 is a cytokine that is primarily produced by various CD4^+^ T cell subsets, such as Th9, Th17, natural killer T cells, follicular helper T cells and some CD8^+^ T cells, but not Tregs (Spolski and Leonard 2014). Previous studies demonstrate that IL‐21 inhibits Treg differentiation and hampers Treg homeostasis (Attridge et al. 2012; Bucher et al. 2009). The graphical summary of the bioinformatics ingenuity pathway analysis (IPA) of the 709 upregulated genes (Figure 3b) identified enrichments of several representative activated regulators, including STAT3, which is a crucial downstream signalling component of IL‐21 (Habib et al. 2003). Moreover, the *Il21*‐centred molecular network demonstrated the involvement of IL‐21 in ‘T helper cell differentiation’, ‘JAK1 and JAK3 in *γ*c cytokine signaling’ and ‘STAT3 pathway’ (Figure 3c). Next, we further confirmed the mRNA and protein levels of IL‐21 in Tregs were dramatically increased, following LPS‐EC‐EVs exposure (Figure 3d). Furthermore, the intracellular IL‐21 staining (Figure 3e,f) validated the observation that LPS‐EC‐EVs activated IL‐21 production in Tregs *in vitro*. Moreover, to investigate the direct effects of EVs on Treg differentiation and function, we isolated splenic naïve CD4^+^ T cells from Foxp3^YFP/Cre^ mice and polarized them into Tregs in the presence of either EC‐EVs or LPS‐EC‐EVs. Strikingly, confocal microscopy analysis demonstrated significant colocalization of Foxp3 and IL‐21 in Tregs following LPS‐EC‐EVs exposure (Figure 3g). IL‐21 has been reported to induce persistent STAT3 phosphorylation to counteract Foxp3 transcription (Goodman et al. 2011). On immunofluorescence and western blotting analysis (Figure 3h,i), STAT3 was heavily phosphorylated and then translocated into the nucleus in Tregs after LPS‐EC‐EVs exposure. Taken together, these results revealed that LPS‐EC‐EVs activated IL‐21/STAT3 signalling in Tregs.

### 3.5 Inhibition of IL‐21 Signalling Reverses EV‐Induced Treg Restriction

Given the significant activation of the IL‐21 signalling pathway in Tregs primed with LPS‐EC‐EVs, we first investigated the effect of IL‐21 deficiency on ALI susceptibility after intratracheally injection of a lethal LPS dose. Compared with wild type (WT) mice, the *Il21* ^−/−^ mice had higher survival rates under LPS challenge (Figure S14a). Consistently, lung histology revealed accelerated repair in survived *Il21* ^−/−^ mice than in survived WT mice (Figure S14b). Taken together, our results demonstrated a potential pathogenic impact of IL‐21 on ALI/ARDS progression and prognosis.

**FIGURE 4:**
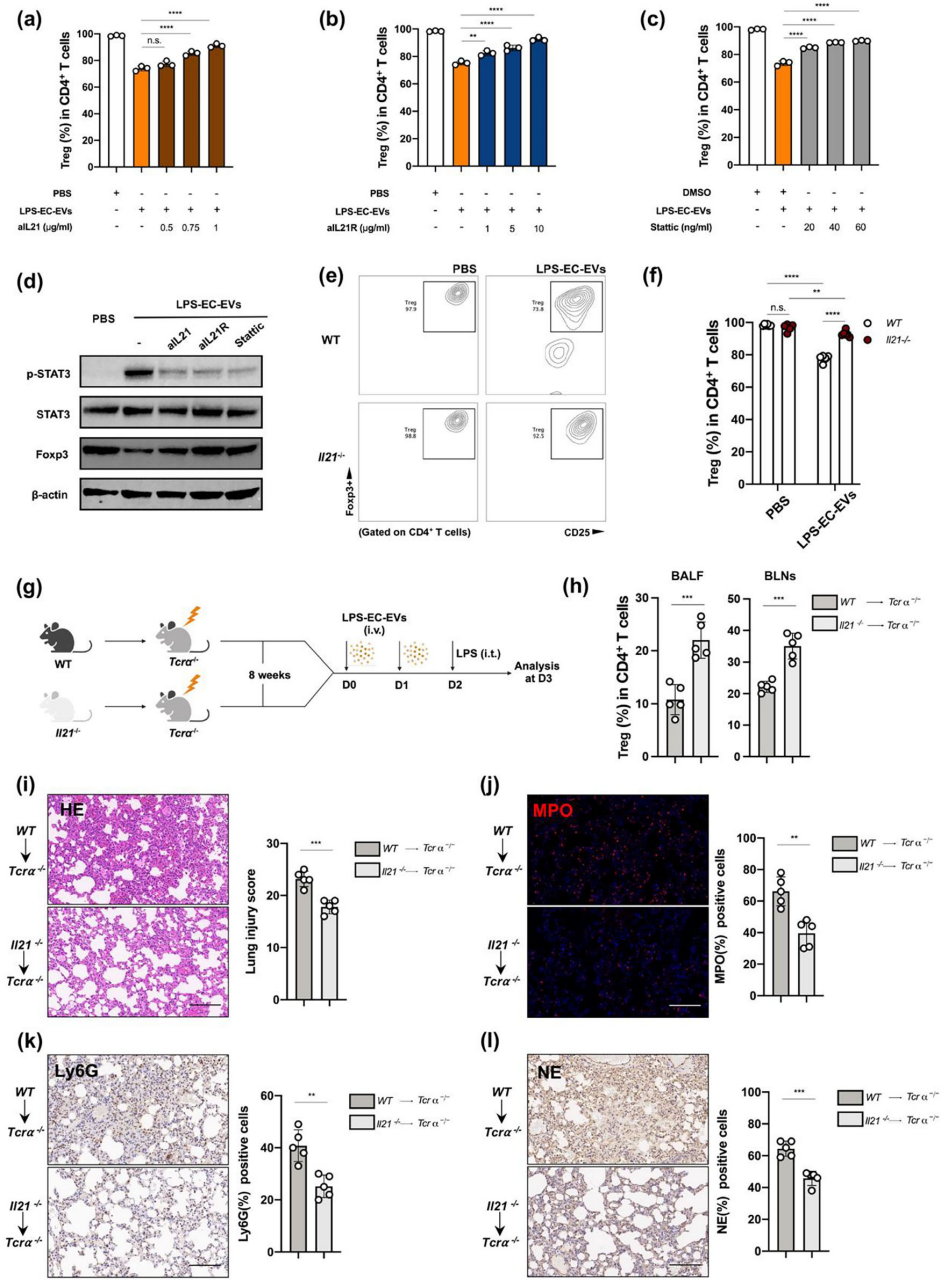
IL‐21 signalling blockade ameliorates the EV‐induced Treg suppression. aIL‐21 (a), aIL‐21R (b) and Stattic (c) were seen to rescue EV‐induced Treg suppression in a dose‐dependent manner (*n* = 3). (d) Immunoblotting of the phosphor‐STAT3 and STAT3 levels of Tregs exposed to LPS‐EC‐EVs after treatment with IL‐21 signalling inhibitors for 5 days (*n* = 3). (e and f) Splenic naïve CD4^+^ T cells were isolated from WT and *Il21^−/−^* mice and then polarized to Tregs with LPS‐EC‐EVs for 5 days. The percentage of Tregs were analyzed via FCM (e) and data were statistically analyzed (f), (*n* = 6). (g) Schematic diagram of ALI induction in chimeras (created using Biorender.com). (h) Quantification of Tregs in BALF and BLNs from different chimeras via FCM (*n* = 5). (i) Representative HE stained tissue sections and lung injury score evaluation (*n* = 5 in each group). Scale bar, 100 µm. (j) Immunofluorescence staining and quantitative analysis of MPO (*n* = 5 in each group). Scale bar, 100 µm. (k and l) Immunohistochemical staining and quantitative analysis of Ly6G (k) and NE (l) (*n* = 5 in each group). Scale bar, 100 µm. **p < 0.01, ***p < 0.005, ****p < 0.001. See also Figure S14.

Next, we examined the effect of IL‐21‐STAT3 axis interrupting on the restricted Treg polarization by LPS‐EC‐EVs *in vitro*. Given that IL‐21 activates STAT3 through its specific heterodimeric receptor (IL‐21R), we used IL‐21 neutralizing monoclonal antibody (NmAb) (aIL21), IL‐21R NmAb (aIL21R) and the STAT3‐specific activation inhibitor Stattic to interrupt this signalling pathway, respectively. The results revealed dose‐dependent restoration of Tregs by IL‐21 NmAb, IL‐21R NmAb and Stattic (Figure 4a–c) when exposed to LPS‐EC‐EVs. Moreover, blockade of IL‐21 signalling significantly inhibited STAT3 hyperphosphorylation, thereby, recovering Foxp3 expression (Figure 4d). Next, naïve CD4^+^ T cells were isolated from *Il21* ^−/−^ mice and polarized into Tregs with LPS‐EC‐EVs. As predicted, the inhibitory effect of LPS‐EC‐EVs was significantly less in *Il21* ^−/−^ Tregs than in WT Tregs (Figure 4e,f).

To further confirm the T cell‐intrinsic effect of IL‐21, we generated chimeric mice by injecting bone marrow (BM) cells from WT or *Il21* ^−/−^ mice into sublethal‐irradiated T‐cell receptor α (*Tcrα*) ^−/−^ mice. These reconstituted mice were intratracheally administered with LPS to induce ALI after two times of LPS‐EC‐EVs pre‐challenge (Figure 4g). The proportions of Tregs in BALF and BLNs were significantly higher in the *Il21* ^−/−^ chimeric mice than in the WT chimeric mice (Figure 4h). Moreover, the severity of pulmonary injury was evaluated by haematoxylin and eosin (HE) staining, and was found to be less in *Il21* ^−/−^ chimeric mice than in the WT chimeric mice (Figure 4i). Considering that Tregs promote neutrophil clearance to control lung inflammation during ALI (Lin et al. 2018), we next examined the levels of MPO, Ly6G and neutrophil elastase (NE), which are indicators of neutrophil function and activation. Consistent with lung injury severity, neutrophil infiltration was less in *Il21* ^−/−^ chimeric mice than in the WT chimeric mice (Figure 4j–l). Taken together, our results demonstrated that LPS‐EC‐EVs suppressed Treg differentiation through a T cell‐intrinsic IL‐21 signalling loop.

### 3.6 Med1 Enriched in LPS‐EC‐EVs Serves as an IL‐21 Transcriptional Regulator

To verify the crucial roles of LPS‐EC‐EV proteins in Treg suppression, we first used 0.1% Triton‐100 and proteinase K to deplete the proteins in LPS‐EC‐EVs. Protein‐depleted LPS‐EC‐EVs showed no significant change in shape and diameter (Figure S15a,b), and depletion efficiency was confirmed by Coomassie Blue staining (Figure S15c). After protein depletion, the LPS‐EC‐EVs had no effect on Treg polarization (Figure S15d,e), implying the important roles of EV proteins in Treg suppression. To further identify which proteins in LPS‐EC‐EVs exerted the function, we reanalyzed the data from our previous experiment (Wang et al. 2024) using unbiased quantitative proteomics by mass spectrometry. There were 136 selectively enriched proteins in LPS‐EC‐EVs, compared with those in EC‐EVs, but was absent of IL‐21 in LPS‐EC‐EVs (Figure S15f) which excluded the possibility that IL‐21 was delivered by LPS‐EC‐EVs directly. In addition, IPA core analysis showed that these upregulated proteins targeted molecules associated with various biological processes in inflammation and immune response (Figure S15g).

**FIGURE 5:**
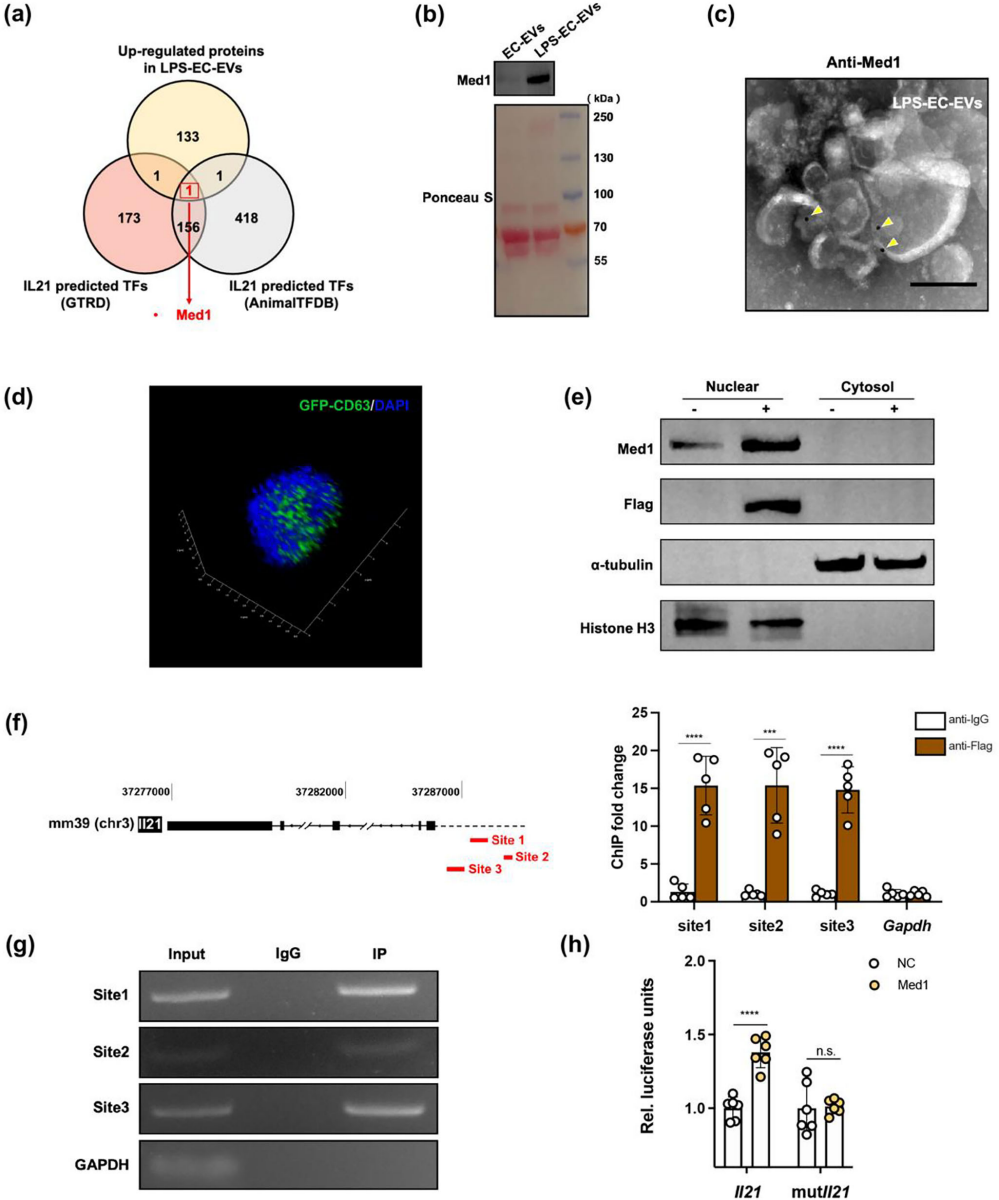
Med1 in LPS‐EC‐EVs serves as an IL‐21 activating TF. (a) Venn diagram of upregulated proteins in LPS‐EC‐EVs and IL‐21 predicted TFs showing that only Med1 was accumulated. (b) Immunoblotting combined with Ponceau S staining confirmed that Med1 was selectively enriched in LPS‐EC‐EVs rather than EC‐EVs (*n* = 3). (c) Representative IEM image showing the presence of Med1 in LPS‐EC‐EVs. Scale bar, 100 nm. (d) 3D view indicated the GFP‐labelled EVs in the Treg nucleus (blue) after coculturing. (e) Immunoblotting analysis confirmed that Med1 was transferred into the Treg nucleus through EVs. Tregs cocultured with Flag‐Med1 LPS‐EC‐EVs and proceeded to isolate nuclear and cytoplasmic fractions (*n* = 3). (f) Schematic model of the primer targeting sites (left) and Flag‐tagged ChIP‐qPCR analysis (right) demonstrated attachment of Med1 to the predicted region on IL‐21 (*n* = 5). (g) Gel‐based imaging of Flag‐tagged ChIP‐qPCR products from (f). (h) Dual‐luciferase assays were performed to confirm the interaction between Med1 and IL‐21 (*n* = 6). ***p < 0.005, ****p < 0.001. See also Figures S15 and S16.

Transcription factors (TFs) are proteins that bind to DNA sequences to turn specific gene transcription on or off. Previous studies reveal that TFs can be sorted into EVs to modulate gene transcription and translation in recipient cells (Ung et al. 2014; Zhou et al. 2018). Therefore, we hypothesized that some LPS‐EC‐EV proteins might serve as TFs to enhance IL‐21 transcription. First, we used two TF prediction databases (GTRD and AnimalTFBD) to combine the enriched LPS‐EC‐EV proteins and screened for potential candidates. Only one protein, Med1, was found (Figure 5a). Med1 expression was confirmed to be dramatically enriched in LPS‐EC‐EVs but not in EC‐EVs by western blotting combined with Ponceau staining (Figure 5b), and immunoelectron microscopy (IEM) (Figure 5c). TFs need to be imported into the nucleus to activate or inhibit transcription. Next, we used GFP labelling to trace the distribution of LPS‐EC‐EVs in Tregs. After 2 days of co‐culture, GFP‐labelled LPS‐EC‐EVs localized within the nucleus, indicating that the protein cargoes in EVs was able to enter the nucleus to interact with DNA (Figure 5d). To further verify that Med1 in LPS‐EC‐EVs entered the nucleus of Tregs, we developed a Med1‐overexpressing EC line with a fusion 3× Flag tag. Flag‐Med1 EVs were collected from these cells after LPS challenge, and then co‐cultured with Tregs for 5 days (Figure S16a,b). Treg subcellular fractionation analysis showed that Flag‐Med1 was enriched in the nuclear fraction (Figure 5e). Taken together, these findings indicated that Med1 in LPS‐EC‐EVs could be delivered into the nucleus of Tregs.

To explore whether Med1 in LPS‐EC‐EVs regulated IL‐21 expression directly in Tregs, we first polarized naïve CD4^+^ T into Tregs in the presence of Flag‐Med1 containing LPS‐EC‐EVs for 5 days. Then we employed Flag‐tagged chromatin immunoprecipitation (ChIP)‐qPCR to analyze Med1 binding at predicted sites within the IL‐21 promoter region in Tregs. The predicted binding sites were found to be in the 2‐kb sequence upstream of the transcription start site of the IL‐21 gene. Our results showed that Flag‐Med1 in EVs bound to all the predicted binding sites (Figure 5f,g). Next, we assessed the impacts of Med1 on IL‐21 gene transcription using a dual‐luciferase reporter assay in HEK293T cells. Compared with the effect in the vector group, Med1 significantly increased the luciferase activity of the IL21‐Luc reporter, and this effect was abolished by mutations in the predicted binding sites (Figure 5h). Our results suggested that enriched Med1 in LPS‐EC‐EVs was an activating TF of IL‐21.

### 3.7 Med1 in LPS‐EC‐EVs Is the Key Molecule That Suppress Tregs

**FIGURE 6:**
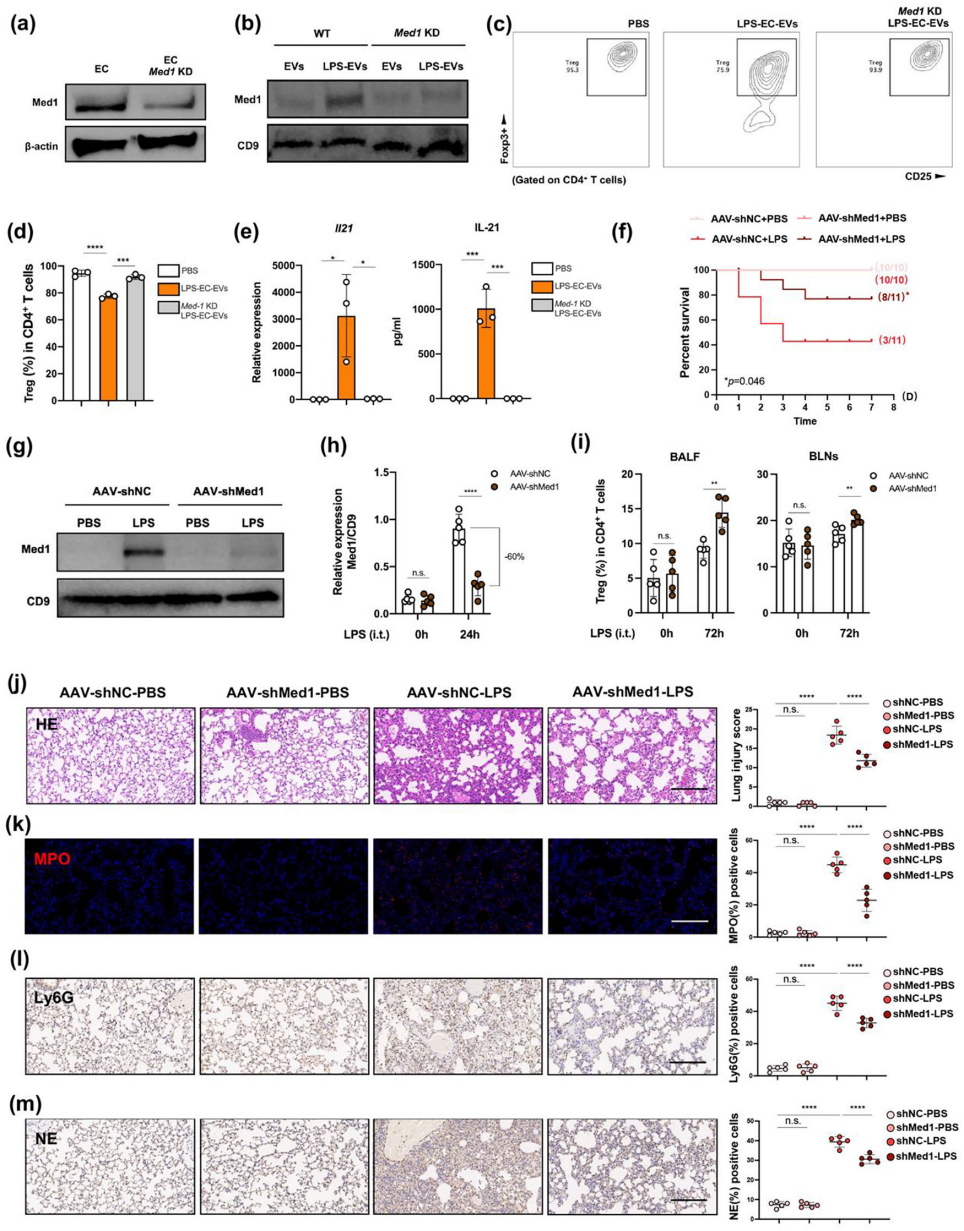
Med1 in LPS‐EC‐EVs is the key modulator for Treg restriction. (a) Immunoblotting showed the Med1 expression in WT and Med1 knockdown ECs (*n* = 3). (b) Med1 depletion efficiency in EVs from WT and Med1 knockdown ECs (*n* = 3). FCM (c) and quantification (d) of CD25^+^Foxp3^+^ double staining in Tregs exposed to Med1‐KD LPS‐EC‐EVs (*n* = 3). (e) RT‐qPCR (left) and ELISA (right) of IL‐21 expression in Tregs and cell culture supernatants (*n* = 3). (f) Seven‐day survival analysis of LPS‐IT mice in shMed1‐AAV‐LungX group and shNC‐AAV‐LungX group. Immunoblotting (g) and quantification (h) of Med 1 expression in BALF‐EVs after AAV‐shNC or AAV‐shMed1 infection (*n* = 5). (i) Quantification of Tregs in BALF and BLNs from different group mice via FCM at 72 h after LPS administration (*n* = 5). (j) Representative HE stained tissue sections and lung injury score evaluation (*n* = 5 in each group). Scale bar, 100 µm. (k) Immunofluorescence staining and quantitative analysis of MPO (*n* = 5 each group). Scale bar, 100 µm. (l and m) Immunohistochemical staining and quantitative analysis of Ly6G (l) and NE (m) (*n* = 5 each group). Scale bar, 100 µm. *p<0.05, **p < 0.01, ***p<0.005, ****p < 0.001. See also Figure S17.

To further explore whether Med1 in LPS‐EC‐EVs was the key substance in the regulation of Tregs, we generated a Med1 knockdown (KD) pulmonary EC line by transduction of a short hairpin RNA targeting Med1 (shMed1) recombinant lentivirus, and the KD efficiency in cells and EVs was evaluated by western blotting (Figure 6a,b). Next, we explored the role of Med1‐KD LPS‐EC‐EVs on Treg differentiation *in vitro*. Compared with LPS‐EC‐EVs, Med1‐KD LPS‐EC‐EVs had minimal effects on Treg reduction (Figure 6c,d) and IL‐21 expression (Figure 6e).

To determine the effect of Med1 in EVs on Treg population *in vivo*, C57BL/6 mice were intravenously injected with a shMed1 pseudotyped adeno‐associated virus LungX (shMed1‐AAV‐LungX) to reduce Med1 expression in pulmonary ECs specifically or inject with shNC‐AAV‐LungX as controls (Figure S17a–c). LPS‐IT mice in the shMed1‐AAV‐LungX group showed significantly higher survival rate than the control group (Figure 6f). We also found that Med1 levels in the BALF‐EVs from LPS‐IT mice was significantly lower in the shMed1‐AAV‐LungX group than in the control group (Figure 6g,h), indicating that pulmonary ECs were the main source of EVs carrying Med1 in BALF. Moreover, the Treg percentage in BALF and BLNs from LPS‐IT mice was significantly higher in the shMed1‐AAV‐LungX group than in the shNC‐AAV‐LungX group, suggesting that pulmonary EC‐derived EVs, carrying Med1, effectively suppressed Treg induction (Figure 6i). Further evaluation showed that compared with the shNC‐AAV‐LungX group, the shMed1‐AAV‐LungX group exhibited less severe lung injury and lower MPO, Ly6G and NE expressions in the infiltrating neutrophils (Figure 6j–m). Taken overall, these data elucidated the pathogenic role of Med1 in pulmonary EC‐derived EVs in inhibiting Treg induction during ALI/ARDS.

### 3.8 Med1 is Accumulated in BALF‐EVs in Patients With ARDS

**FIGURE 7:**
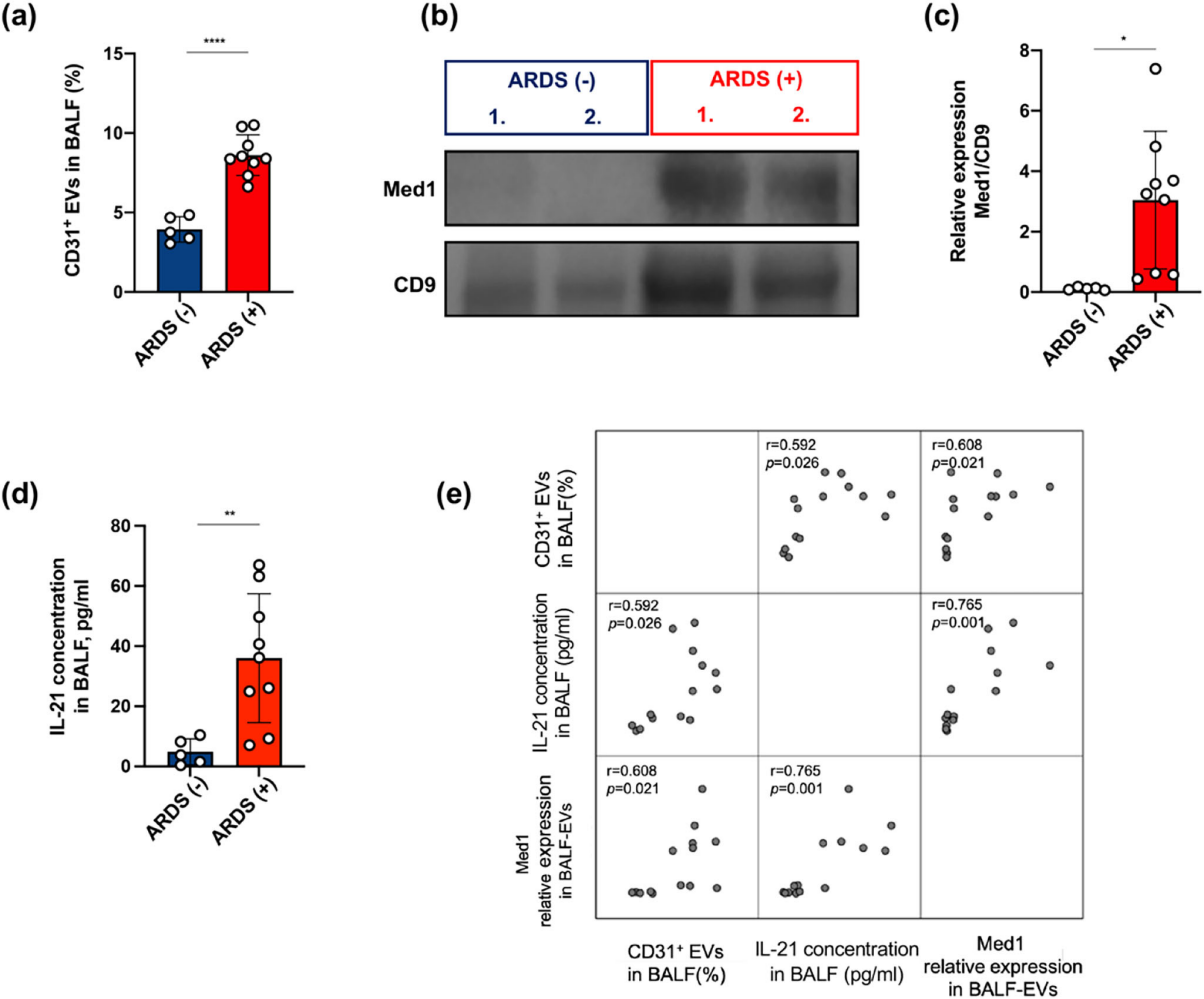
Elevated Med1 expression in BALF‐EVs from ARDS patients. (a) Comparison of CD31^+^ EVs percentage in the BALF from non‐ARDS (*n* = 5) and ARDS (*n* = 9) patients. Immunoblotting (b) and quantification (c) of Med1 expression in BALF‐EVs from non‐ARDS (*n* = 5) and ARDS patients (*n* = 9). (d) IL‐21 concentrations in the BALF from non‐ARDS (*n* = 5) and ARDS (*n* = 9) patients. (e) Correlation analysis of different variables including CD31^+^ EV proportion, Med1 relative expression in BALF‐EVs and IL‐21 concentration in BALF. *p < 0.05, **p < 0.01, ****p < 0.001. See also Figure S18.

Finally, we conducted a cohort discovery study recruiting nine patients with ARDS and five postoperative patients on mechanical ventilation as controls. The patient characteristics were shown in Table S2. EVs were isolated from the BALF samples of patients by ultracentrifugation and identified by capture beads‐based FCM (Figure S18). Compared with the control group, the patients with ARDS had significantly higher proportion of BALF CD31^+^ EVs and Med1 accumulation (Figure 7a,b). Moreover, the expression of Med1 normalized to the EV loading control CD9 was significantly higher in patients with ARDS than in the control group (Figure 7c). In addition, the concentrations of IL‐21 in the BALF samples derived from ARDS patients were significantly higher than those derived from non‐ARDS patients (Figure 7d). Besides, we observed that the relative expression of Med1 in BALF‐EVs showed significant correlations with CD31^+^ EV proportion and IL‐21 concentrations in BALF (Figure 7e). Taken together, these results demonstrated that Med1 expression in BALF‐EVs might be a potential biomarker in ARDS patients.

## 4 Discussion

Tregs comprise only 5%–20% of the total number of pulmonary CD4^+^ T cells but exert vital and classical roles in regulating lung inflammation (Jovisic et al. 2023). Reduced number of Tregs contributes to progressive tissue damage and death in ARDS. Adoptive transfer of Tregs into experimental ALI mice (D'Alessio et al. 2009) and patients with COVID‐19‐associated ARDS (Gladstone et al. 2020) are found to ameliorate lung injury and improve outcomes. Understanding the role of the microenvironment in Treg dysfunction in ALI/ARDS may provide clues to the therapeutic targets. In this study, we provide hitherto unknown evidences that EVs derived from activated pulmonary ECs suppress Tregs in an IL‐21‐dependent manner, which stimulates STAT3 hyperphosphorylation to counteract Foxp3 transcription. We characterize the proteomics of LPS‐EC‐EVs and find that Med1 in EVs acts as an IL‐21 TF. These results indicate the involvement of pulmonary endothelial‐derived EVs in the development of an overwhelming inflammatory ALI/ARDS microenvironment by suppressing Tregs directly.

Tregs are the main cells that support tissue homeostasis and self‐tolerance by controlling overactive immune responses. Insufficient or impaired Tregs can contribute to the onset and worsening of multiple inflammatory and autoimmune diseases. However, excessive Treg accumulation is associated with overtly immunosuppressive conditions, which contribute to chronic infections and cancer development (Shan et al. 2022; Herzmann et al. 2012). Activation of naïve CD4^+^ T cells to differentiate into Tregs in the periphery requires additional signals from the microenvironment, along with the TCR and costimulatory receptors (Josefowicz et al. 2012). As important microenvironment components, EVs are able to shape Treg differentiation under various situations. For instance, Kimura et al. (2018) reported that circulating EVs suppress Treg induction in patients with multiple sclerosis. On the other hand, Jiang et al. (2016) documented that intestinal epithelial cell adhesion molecule‐positive EVs are able to induce Tregs to control inflammatory bowel disease (IBD) in a TGF‐β1‐dependent manner. In addition, multiple cancer cell‐derived EVs are reported to promote Treg differentiation to induce tumour immunosuppression (Shen et al. 2020; Mrizak et al. 2014; Wang et al. 2018). In this study, we find that pulmonary CD31^+^ EVs comprise a considerable proportion of the total EVs in the lungs and play crucial roles in regulating adaptive Treg population during ALI/ARDS. Taking into account the findings of this study and our previous studies, we speculate two mechanisms of EVs derived from activated pulmonary ECs in promoting lung inflammation, as follows: (1) direct interaction with proinflammatory cells, such as monocytes and neutrophils, to enhance the production of proinflammatory mediators (Wang et al. 2024; Hu et al. 2022; Zi et al. 2024) and (2) suppression of Tregs to delay resolution of inflammation. Taken together, these results suggest that targeting the release, depletion, or modification of pulmonary endothelial‐derived EVs is a potential treatment strategy for ALI/ARDS.

TGF‐β and IL‐2 signalling are curial for Treg differentiation and maintenance in the periphery. To our surprise, LPS‐EC‐EVs have minimal impacts on these cytokine signalling cascades. Further experiments reveal that LPS‐EC‐EVs suppress Tregs in an IL‐21‐dependent manner. IL‐21 is a type I cytokine that activates various signalling pathways (e.g., JAK‐STAT, PI3K, MAPK and AKT) by binding to IL‐21R and the common γ‐chain subunit. Since its discovery in the year 2000, IL‐21 has been revealed by several studies to be an effective therapeutic target in various immune‐associated diseases, such as rheumatoid arthritis, IBD, psoriasis and systemic lupus erythematosus, owing to its capability to modulate the activities of both immune and nonimmune cells (Monteleone et al. 2009). However, to the best of our knowledge, the potential effect of IL‐21 in ALI/ARDS has remained largely unknown. IL‐21 has been well established to negatively regulate Treg differentiation or stability (Yang et al. 2016; Saxena et al. 2021) by inducing persistent STAT3 phosphorylation and nuclear translocation, even in the presence of TGF‐β and IL‐2. STAT3 is a well‐known inhibitor of Foxp3 expression. In this study, blockade of the IL‐21/IL‐21R/STAT3 signalling pathway counteracts EV‐induced Treg suppression. Moreover, the survival rate is significantly higher in *Il21* ^−/−^ ALI mice than in WT ALI mice. These data indicate that IL‐21 neutralization may be an effective ALI/ARDS treatment by disrupting the interaction between pulmonary ECs and Tregs. Intriguingly, recent reports show that IL‐21 is able to drive Treg apoptosis (Tortola et al. 2019) or pyroptosis (Chang et al. 2023), thereby, inducing Treg loss. However, these phenomena are not observed in our study. EVs carry several functional molecules, such as RNAs, proteins and metabolites, to mediate cell–cell communication. We speculate that some of these cargoes in LPS‐EC‐EVs helps Treg evasion from IL‐21‐induced programmed cell death.

Despite the widely described effects of different RNAs in EVs (Tosar et al. 2021), our present study indicates that proteins in LPS‐EC‐EVs also play important roles and account for the majority of the observed Treg restriction. Analysis of the differentially enriched proteins in LPS‐EC‐EVs shows that they are associated with immune responses, thereby, providing new insights into the interaction of ECs with immune cells through EVs. Among these enriched proteins, we find that Med1 mediates Treg suppression. Unlike other Mediator complex subunits, Med1 is a nonessential subunit that may serve other biological functions. Previous studies identify that Med1 is critical for invariant natural killer T (Yue et al. 2011) and effector CD8^+^ T cell (Jiao et al. 2022) development, and controls thymic T cells migration into lymph nodes (Yuan et al. 2024). However, the roles of Med1 in Treg functions remain elusive. Recently, Chaudhuri et al. (2024) revealed that Med1 is required for a specific effector Treg differentiation program in the tumour microenvironment. They find that Treg‐intrinsic Med1 is required to maintain tumour‐associated immunosuppression, independent of Foxp3 expression. Our work shows that enriched Med1 in LPS‐EC‐EVs can be delivered into the Treg nucleus and hamper Treg stability by activating IL‐21 signalling. Among the Mediator complex components, Med1 has the largest intrinsically disordered region and is able to interact with heterogeneous DNAs as a potential TF (Lyons et al. 2023). High local concentrations of Med1 may alter Treg transcription patterns and induce a transcriptional burst of IL‐21. Combined with the results of the study of Chaudhuri et al., our results highlight the plasticity and complexity of Med1 in orchestrating Treg phenotypes and functions.

The status of the parent cells determines the cargoes within EVs. For example, EVs secreted by apoptotic endothelium carry cytokines (Berda‐Haddad et al. 2011) and adhesion molecules (Combes et al. 1999) to exacerbate inflammation. Our proteomic analysis combined with further experiments reveal that Med1 is highly enriched in BALF‐EVs in both ALI mice and patients with ARDS, and EVs carrying Med1 are mainly derived from pulmonary ECs. Several studies demonstrate the physiological and pathological roles of Med1 in the endothelium. In a mouse pulmonary arterial hypertension model (Wang et al. 2022), Med1 dysregulation impairs endothelial bone morphogenetic protein and TGF‐β signalling pathways, thereby, contributing to disease progression. However, little is known about the involvement of Med1 in endothelium‐mediated inflammation. Our study reveal that activated pulmonary ECs use Med1 as an effector EV cargo to communicate with Tregs and inhibition of Med1 accumulation in these EVs may be a potential therapeutic approach in ALI/ARDS.

Collectively, we have explored the pathogenic roles of EVs from activated pulmonary ECs with high levels of Med1 in Treg suppression and ALI/ARDS progression. We verify that Med1 in these EVs is delivered into Tregs and then activates IL‐21 signalling, which induces Treg instability through sustained STAT3 phosphorylation. Our study expands the understanding of EVs from activated pulmonary ECs and reveals their previously undiscovered roles in ALI/ARDS.

### 4.1 Limitation of this Study

We systematically investigated the role of activated pulmonary endothelial‐derived EVs in ALI/ARDS. However, the present study has some limitations. First, no single animal model can recapitulate the complexity or persistence of human ARDS. Thus, the findings and conclusions should be further confirmed using other type models of lung injury, for example, the bacterial pneumonia models. Second, we defined that CD31^+^ EVs were derived from ECs. However, some immune cells also express CD31 and may secret CD31^+^ EVs. Third, in PBA analysis of BALF‐EVs, some decreased EV subpopulations, such as cluster 3, may have potential roles in regulating inflammation. Furthermore, we collected EVs from the EC culture supernatants *in vitro*, which may differ from the EVs derived from pulmonary ECs *in vivo*. Finally, Med1 is an important coactivator involved in the gene transcription in cells. Knocking down the expression of Med1 in pulmonary ECs may alter the other cargoes in EVs, not only Med1. Thus, a further analysis of Med1 in EVs is needed to explore the role in ALI/ARDS.

## Author Contributions

**Xu Liu**: conceptualization, investigation, writing – original draft, visualization, writing – review and editing, validation, methodology, software, formal analysis, data curation. **Wei Huang**: writing – review and editing, methodology, supervision, data curation, resources, formal analysis. **Xiwen Zhang**: data curation, resources, methodology, conceptualization, formal analysis. **Shiming Li**: methodology. **Haofei Wang**: methodology. **Zongsheng Wu**: methodology. **Shuangfeng Zi**: methodology. **Lu Wang**: methodology. **Ling Liu**: methodology. **Yi Yang**: methodology. **Jianfeng Xie**: conceptualization, investigation, supervision. **Mingzhu Zheng**: conceptualization, investigation, supervision, data curation, writing – review and editing. **Jie Chao**: conceptualization, investigation, writing – review and editing, data curation, supervision. **Haibo Qiu**: conceptualization, funding acquisition, resources, supervision.

## Funding

This work was support by the Key Program of the National Natural Science Foundation of China (No. 81930058), National Science and Technology Major Project (No. 82341032), the National Key Research and Development Program of China (2022YFC2504400, 2022YFC2504405, 2022YFC2304600), the National Natural Science Foundation of China (No. 82202390, No. 82202420) and the Natural Science Foundation of Jiangsu Province (Youth project, BK20220833).

## Conflicts of Interest

The authors declare no conflicts of interest.

## Supporting information




**Supplementary Table**: jev270235‐sup‐0001‐TableS1.xlsx

**Supplementary Table**: jev270235‐sup‐0002‐TableS2.xlsx

**Supplementary Table**: jev270235‐sup‐0003‐TableS3.xlsx

**Supplementary Table**: jev270235‐sup‐0004‐TableS4.xlsx

**Supplementary Figures**: jev270235‐sup‐0005‐Figures.docx

## Data Availability

All data needed to evaluate the conclusion in this paper are all presented in the paper and/or in the supplementary materials.
