# Supplementary material for: Endothelium‐Treg Communication Through Extracellular Vesicle Transfer Exacerbates Acute Respiratory Distress Syndrome
Source: J Extracell Vesicles. 2026 Feb 8;15(2):e70235. doi: 10.1002/jev2.70235 (PMC12884014; doi:10.1002/jev2.70235)
Supplement: Supplementary file 5 — Supplementary Figures: jev270235‐sup‐0005‐Figures.docx [file JEV2-15-e70235-s004.docx]

Supplementary materials

This file contains:

FIGURE S1-18


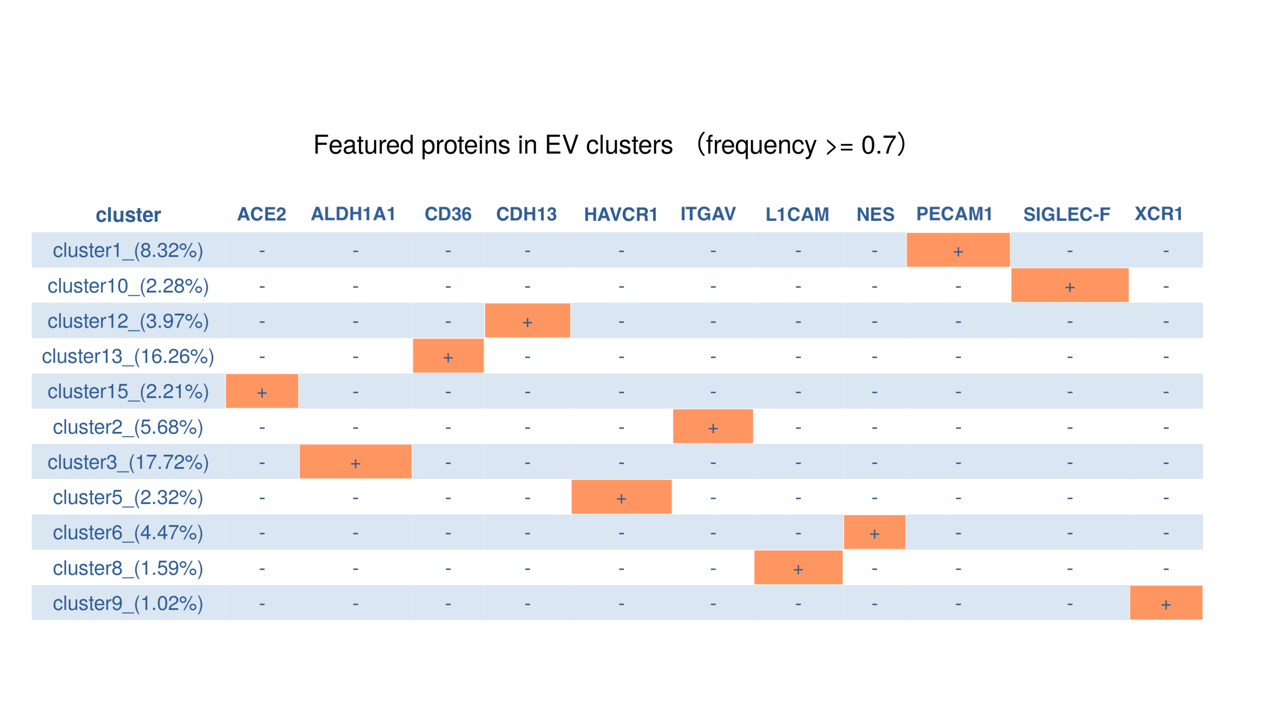


**FIGURE 1** **Feature protein of each BALF EV subpopulation.**


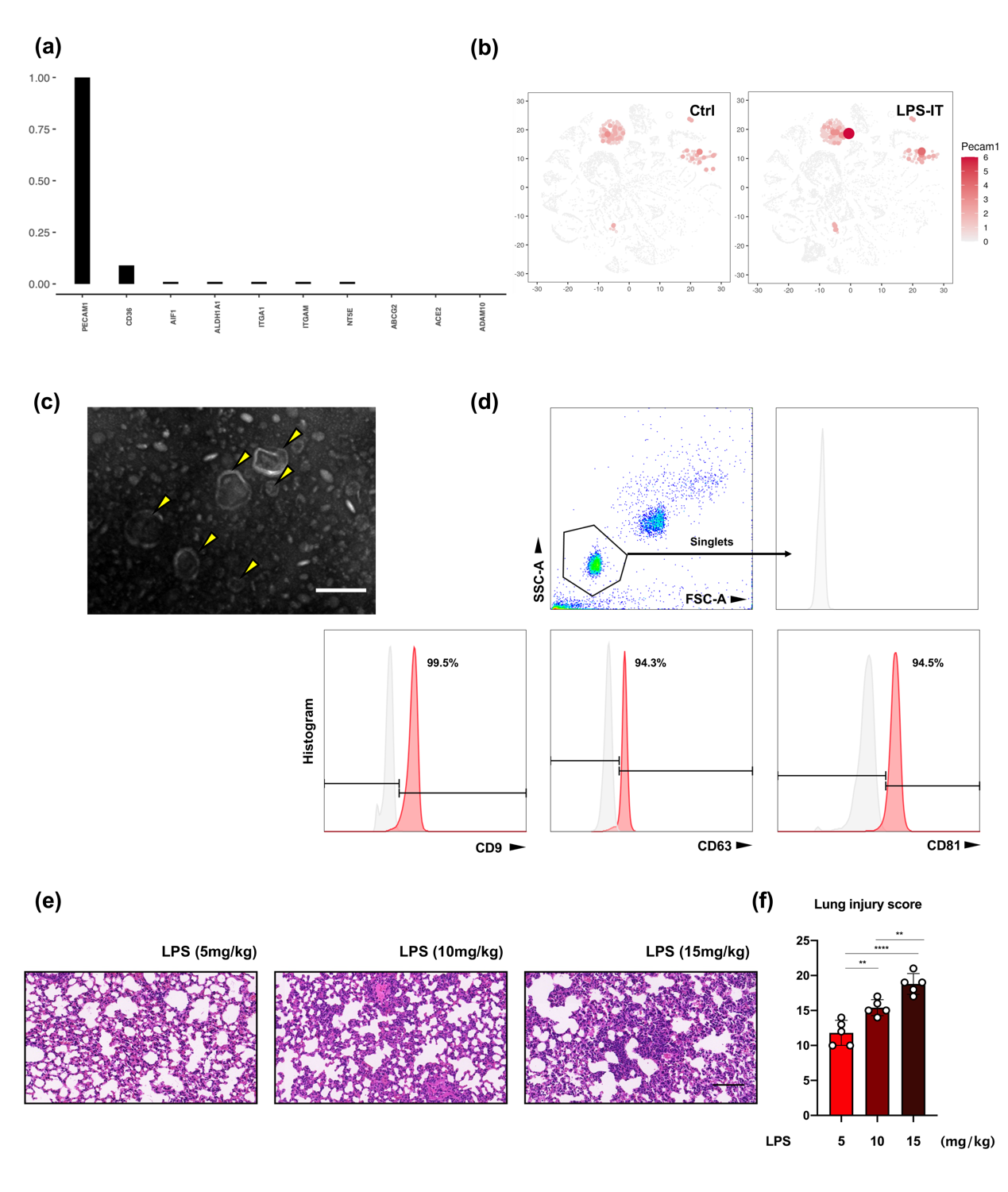


**FIGURE S2** **Characterization of CD31^+^ EVs in the BALF of LPS-IT mice.** (a) The protein constitution of cluster 1. (b) Cluster 1 was higher in LPS-IT mice than in the control mice. (c) Representative TEM image of mouse BALF EVs. Scale bar, 200 nm. (d) The gating strategy of magnetic beads-based FCM for EV surface protein detection (top) and staining of EV surface markers (bottom). (e-f) Representative HE stained tissue sections (e) and lung injury score (f) after administration of different LPS dose. Scale bar, 100 μm.


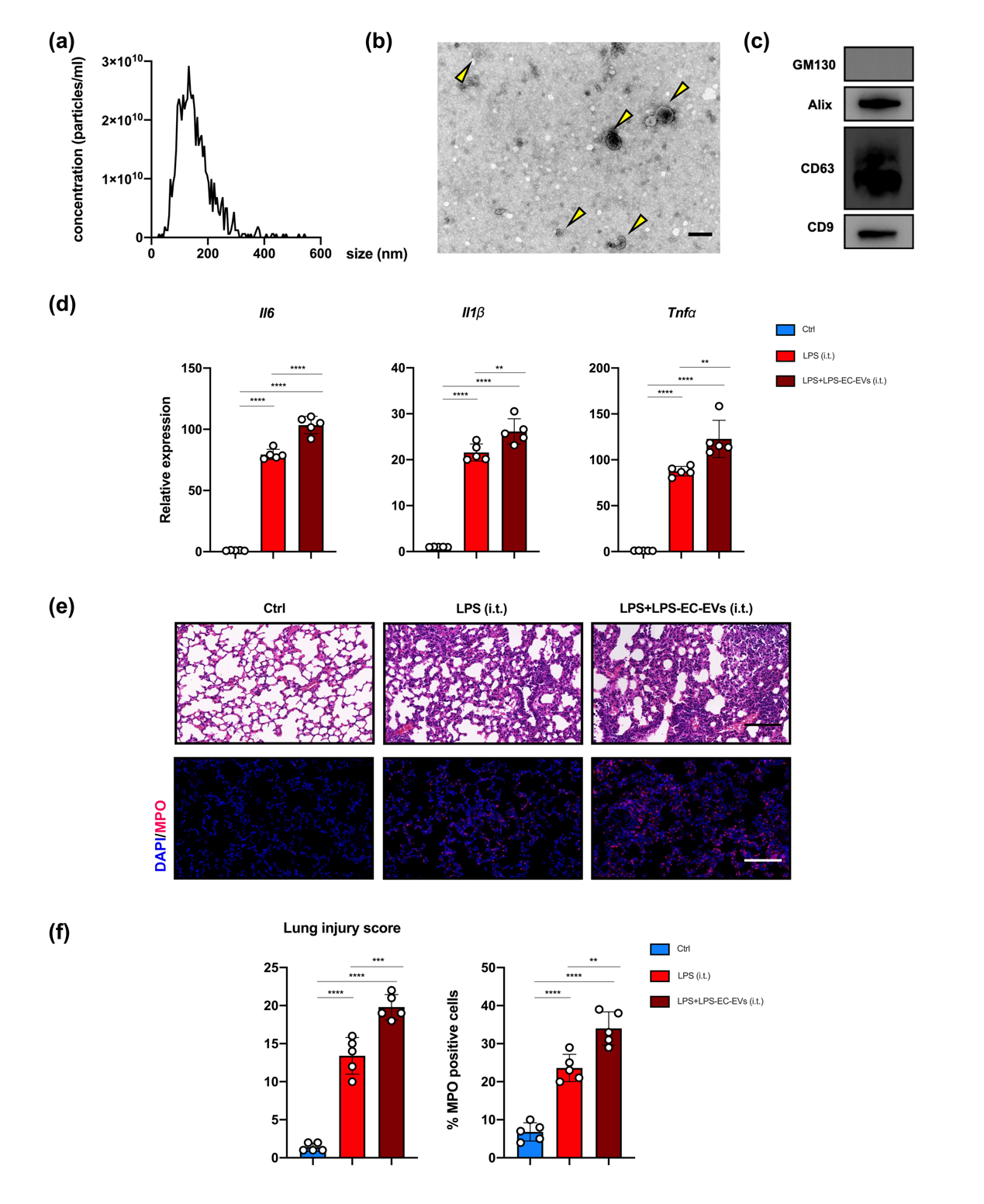


**FIGURE S3** **LPS-EC-EVs aggravate LPS-induced lung inflammation and damage.** (a to c) Identification of isolated EVs from LPS-activated ECs. NTA (a), TEM (b) and immunoblotting of protein biomarkers (c) of EVs were shown. Scale bar, 200 nm. (d) RT-qPCR analysis of inflammatory gene expressions in lung tissues from different group mice (n=5 each group). (e) HE staining (top) and MPO immunofluorescence staining (bottom) of lung tissue sections from different group mice (n=5). Scale bar, 100 μm. (f) Lung injury score and MPO^+^ neutrophil count (n=5). The LPS group of mice was injected intratracheally with 5 mg/kg LPS, whereas the control group mice were injected with the same amount of sterile PBS. For LPS+LPS-EC-EVs, mice were intratracheally administrated with LPS plus 50 μg LPS-EC-EVs. ***p*<0.01, *****p*<0.001.


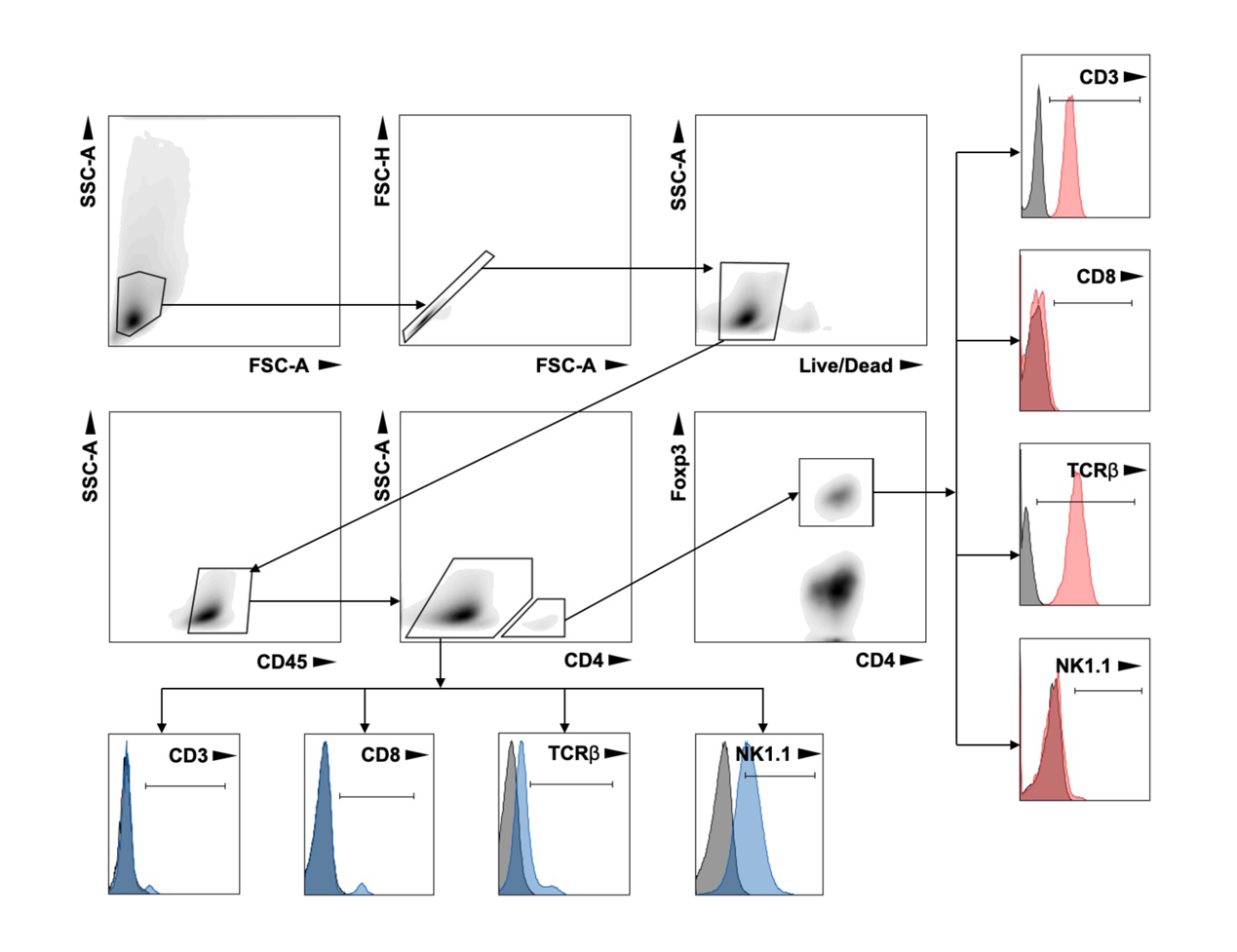


**FIGURE S4** **Gating strategy of Tregs in the BALF and BLNs from** **mice.** The gated Tregs in BALF and BLNs were CD3^+^, TCRβ^+^, while CD8^-^ and NK1.1^-^.


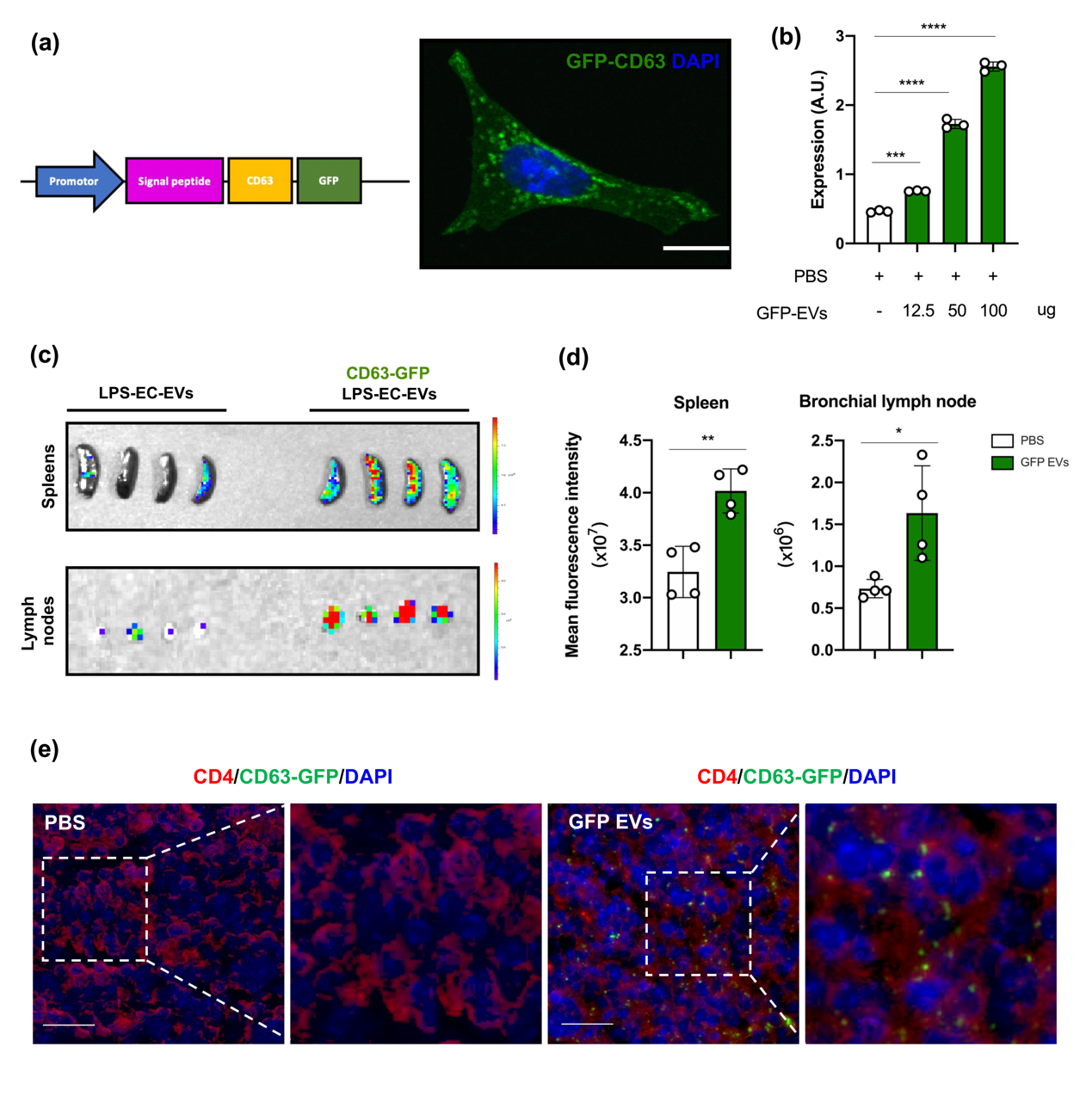


**FIGURE S5** **Accumulation of GFP-labelled LPS-EC-EVs in lymphoid organs.** (a) Schematics diagram of DNA constructs to produce GFP-CD63 proteins and fluorescence image of transduced MPMEC. (b) Quantification of the fluorescence intensity of GFP-labelled LPS-EC-EVs (n=3). (c) Fluorescence images of spleens and BLNs at 24 h after tail vein injection of sterile PBS or GFP-labelled LPS-EC-EVs (100 μg / per mouse, n=4). (d) Quantification of the fluorescence intensity in spleens and BLNs in FIGURE S5c. (e) Immunostaining for detection of CD4 and GFP-labelled EVs in BLNs. Scale bar, 10 μm. **p*<0.05, ***p*<0.01, ****p*<0.005, *****p*<0.001.


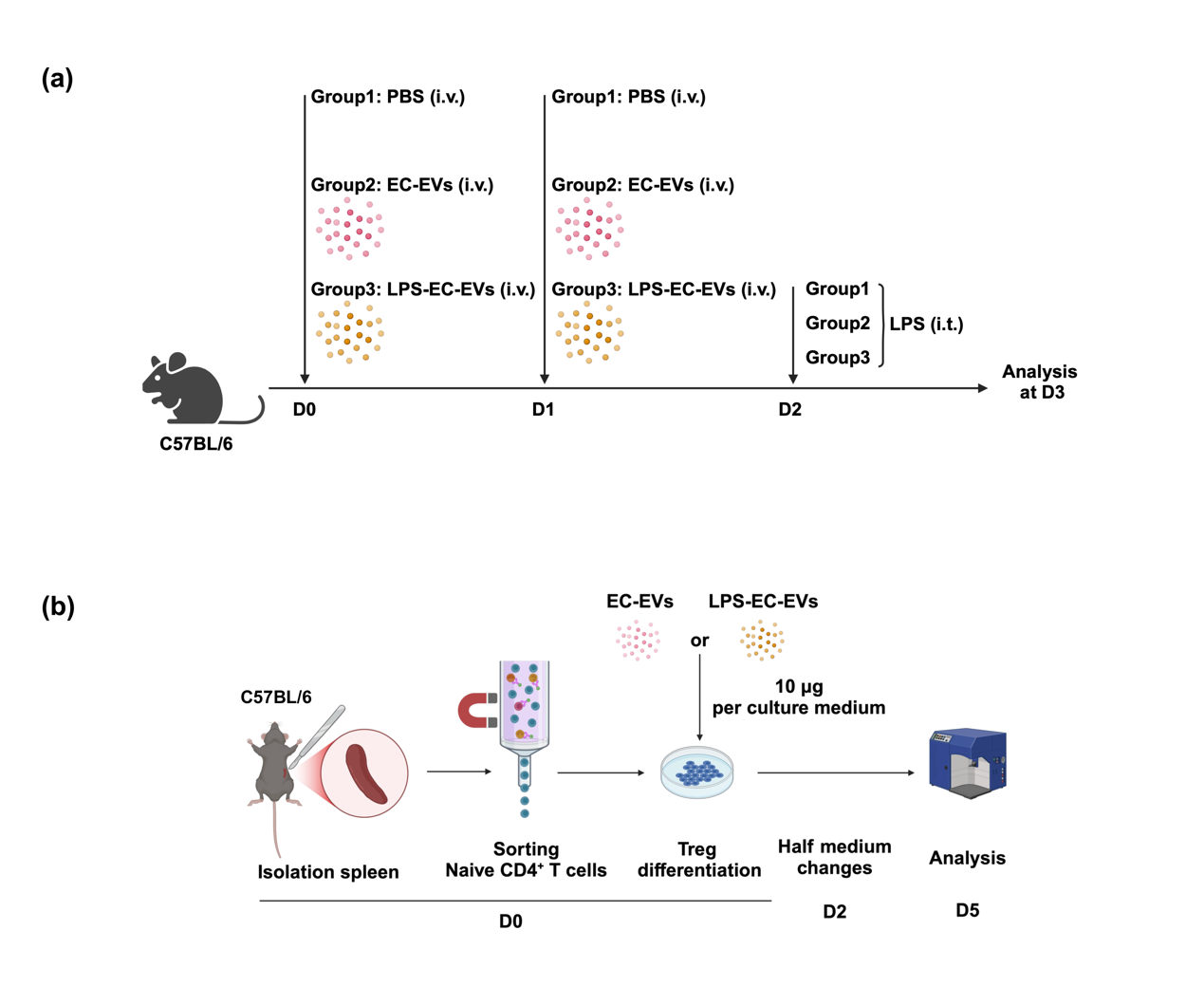


**FIGURE S6 Experimental outlines to investigate the role of EVs on Treg differentiation.** (a) Schema of the experimental ALI model in mice. On day 0 and day 1, mice received either PBS or different EVs (100 μg per mouse) pre-treatments. (b) Experimental setup for *in vitro* Treg analysis. Naïve CD4^+^ T cells were isolated from C57BL/6 mice and polarized into Tregs with either PBS or different EVs (created using Biorender.com).


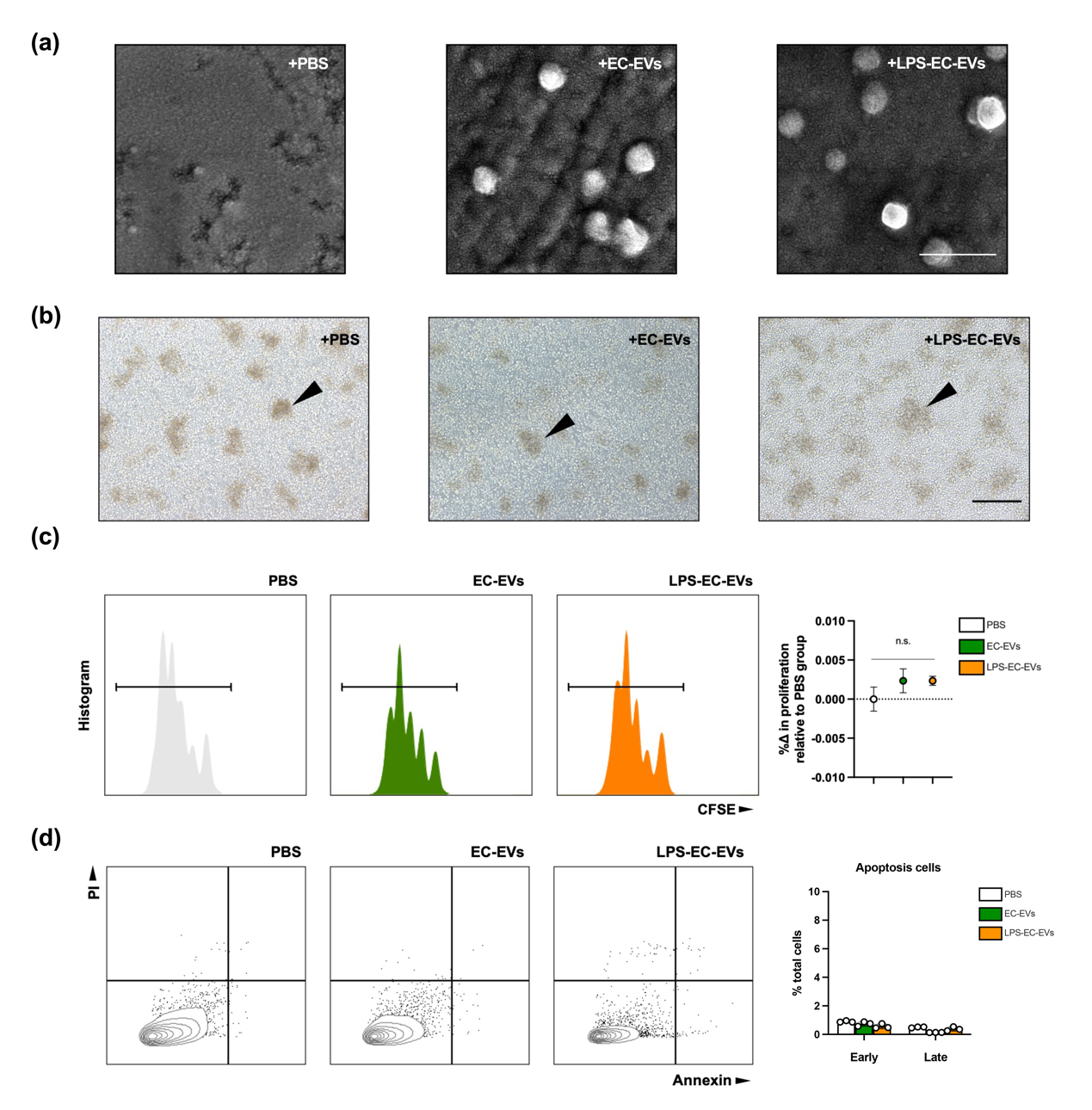


**FIGURE S7 The effects of LPS-EC-EVs on Treg survival, proliferation and apoptosis.** (a) Typical SEM image of Treg surface exposed to either PBS or different EVs for 1 day. Scale bar, 200 nm. (b) Light microscopy images of Tregs exposed to either PBS or different EVs for 5 days (n=3). Scale bar, 200 μm. (c) Representative histograms and quantification of Treg proliferation w/wo EVs by FCM analysis (n=3). %Δ in proliferation = [(proliferation with EVs − proliferation with vehicle)/proliferation with vehicle] x 100. (d) Representative FCM plots and quantification of Tregs apoptosis exposed to either PBS or different EVs for 5 days (n=3).


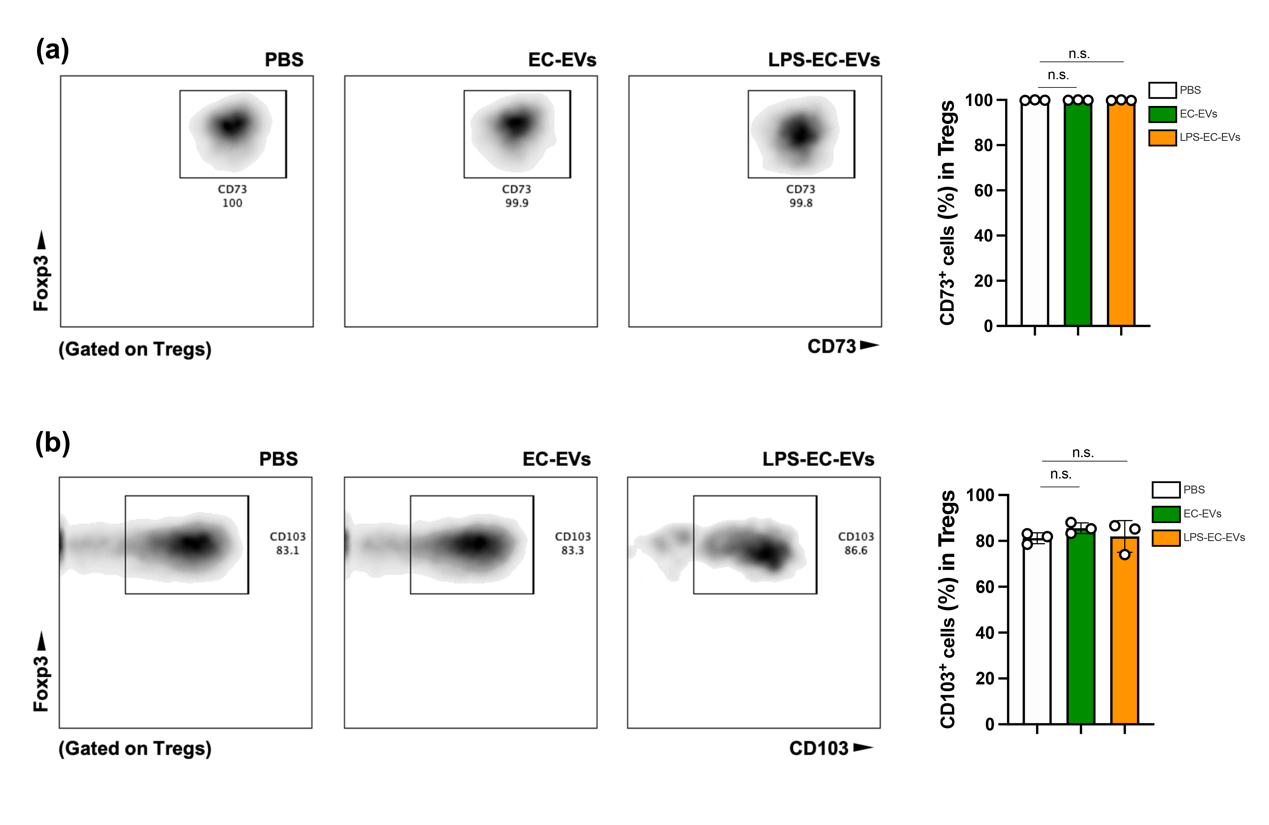


**FIGURE S8 LPS-EC-EVs have no effects on the expression of CD73 and CD103 in Tregs.** (a) FCM analysis of CD73 expression in different group Tregs (n=3); (b) FCM analysis of CD103 expression in different group Tregs (n=3);


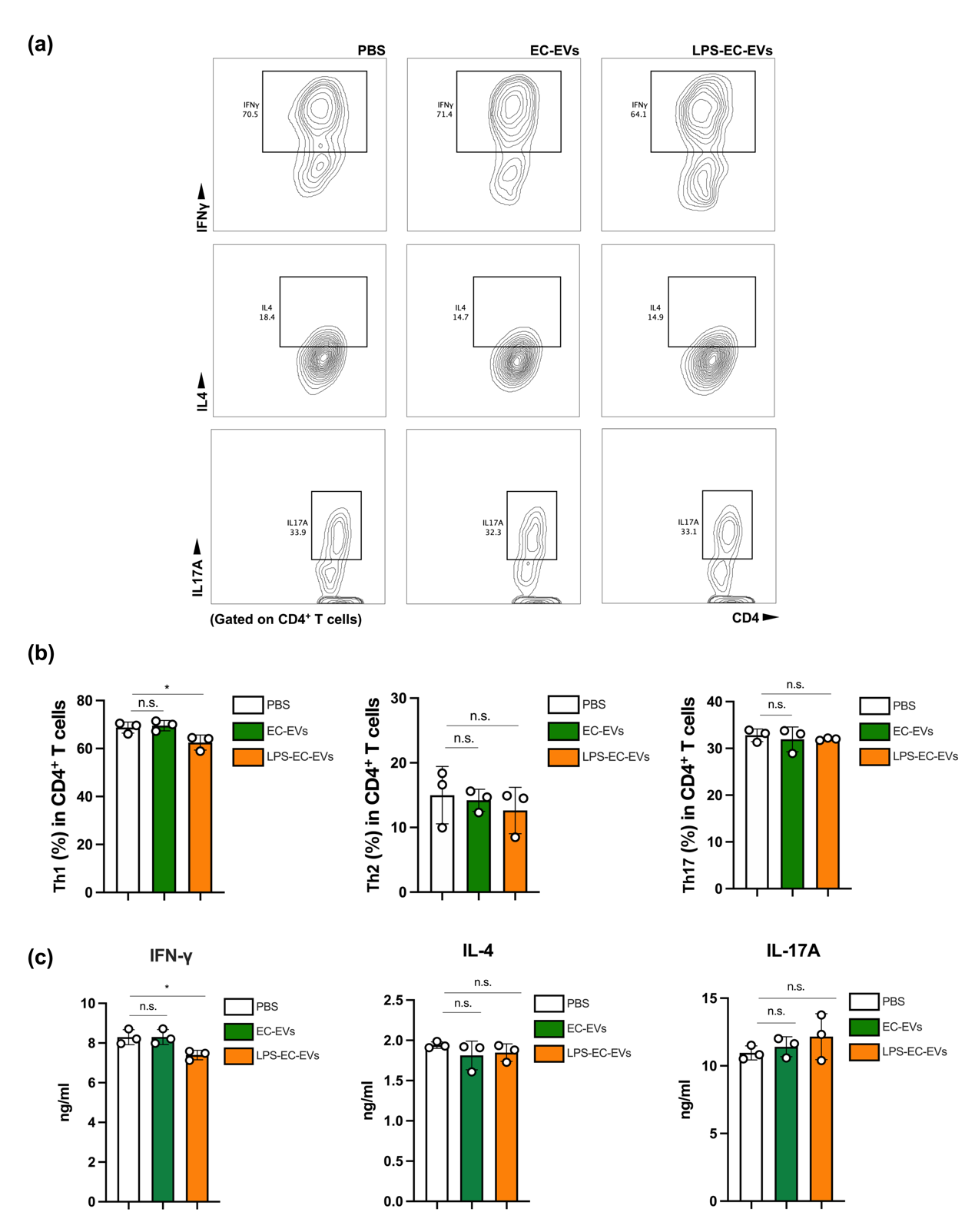


**FIGURE S9 Effects of LPS-EC-EVs on conventional effector T cells (Th1, Th2 and Th17) induction.** (a) FCM and (b) quantification of conventional effector T cells exposed to either PBS or different EVs (n=3). (c) Cytokine concentrations in the culture medium from different groups above (n=3). **p*<0.05.


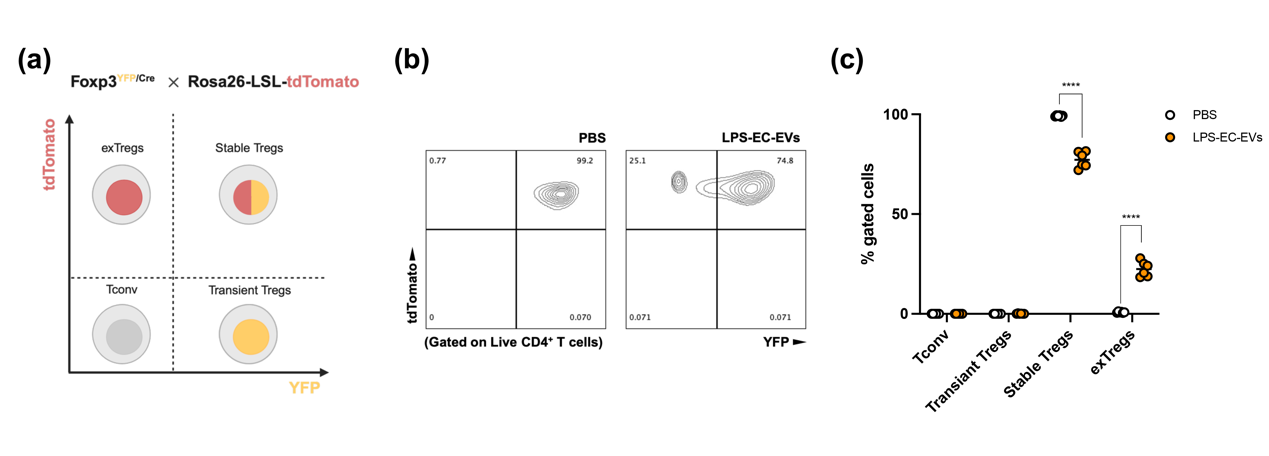


**FIGURE S10 LPS-EC-EVs destabilize Tregs.** (a) Schematic diagram of the generation of Treg-linage tracing mice with fluorescence patterns for conventional T cells (Tconv, YFP^-^tdTomato^-^), transient Tregs (YFP^+^tdTomato^-^), stable Tregs (YFP^+^tdTomato^+^) and exTregs (YFP^-^tdTomato^+^) (created using Biorender.com). (b) Representative FCM plot and (c) quantification of the four cell populations above in each group (n=6).


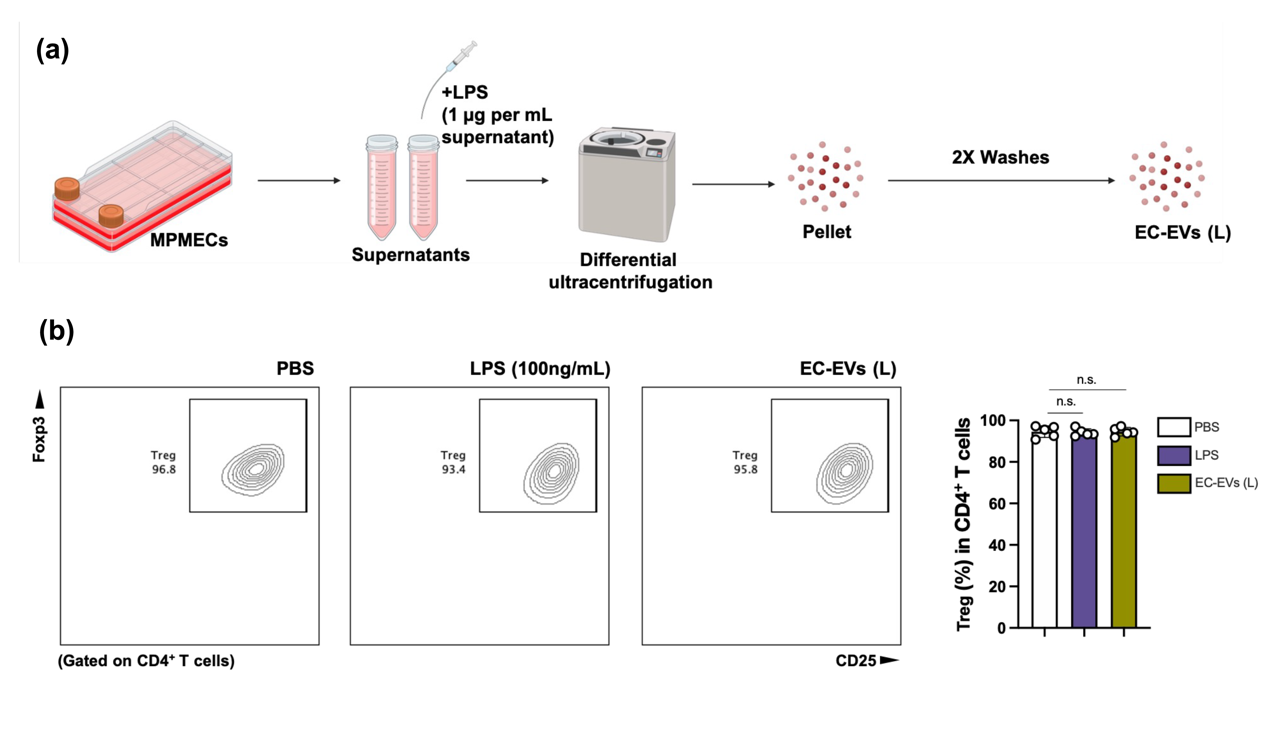


**FIGURE S11 LPS-EC-EVs does not influence naïve CD4^+^ T cells polarization into Tregs.** (a) Schema of EC-EVs (L) preparation (created using Biorender.com). (b) Isolated naïve CD4^+^ T cells were polarized into Tregs with PBS, LPS or EC-EVs (L) for 5 days followed by intracellular Foxp3 staining (n=5).


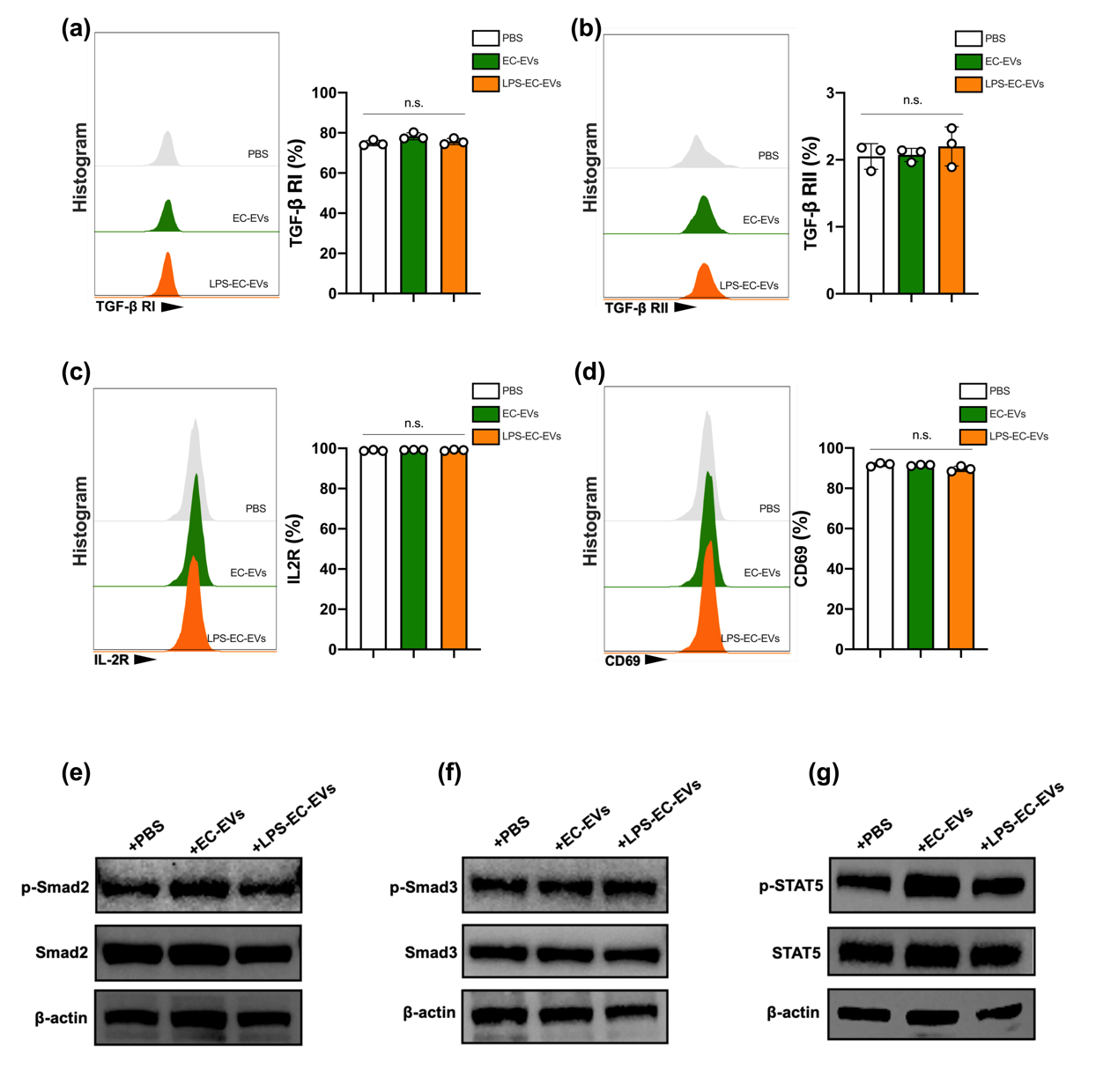


**FIGURE S12 Comparable TGF-β and IL2 signaling pathways after EVs challenge.** (a to d) Histograms and quantification of TGF-β receptor I (a), II (b), CD25 (c) and CD69 (d) on Tregs exposed to PBS or different EVs for 5 days (n=3). (e to g) smad2 (e), smad3 (f) and STAT5 (g) phosphorylation in Tregs exposed to either PBS or different EVs for 5 days (n=3).


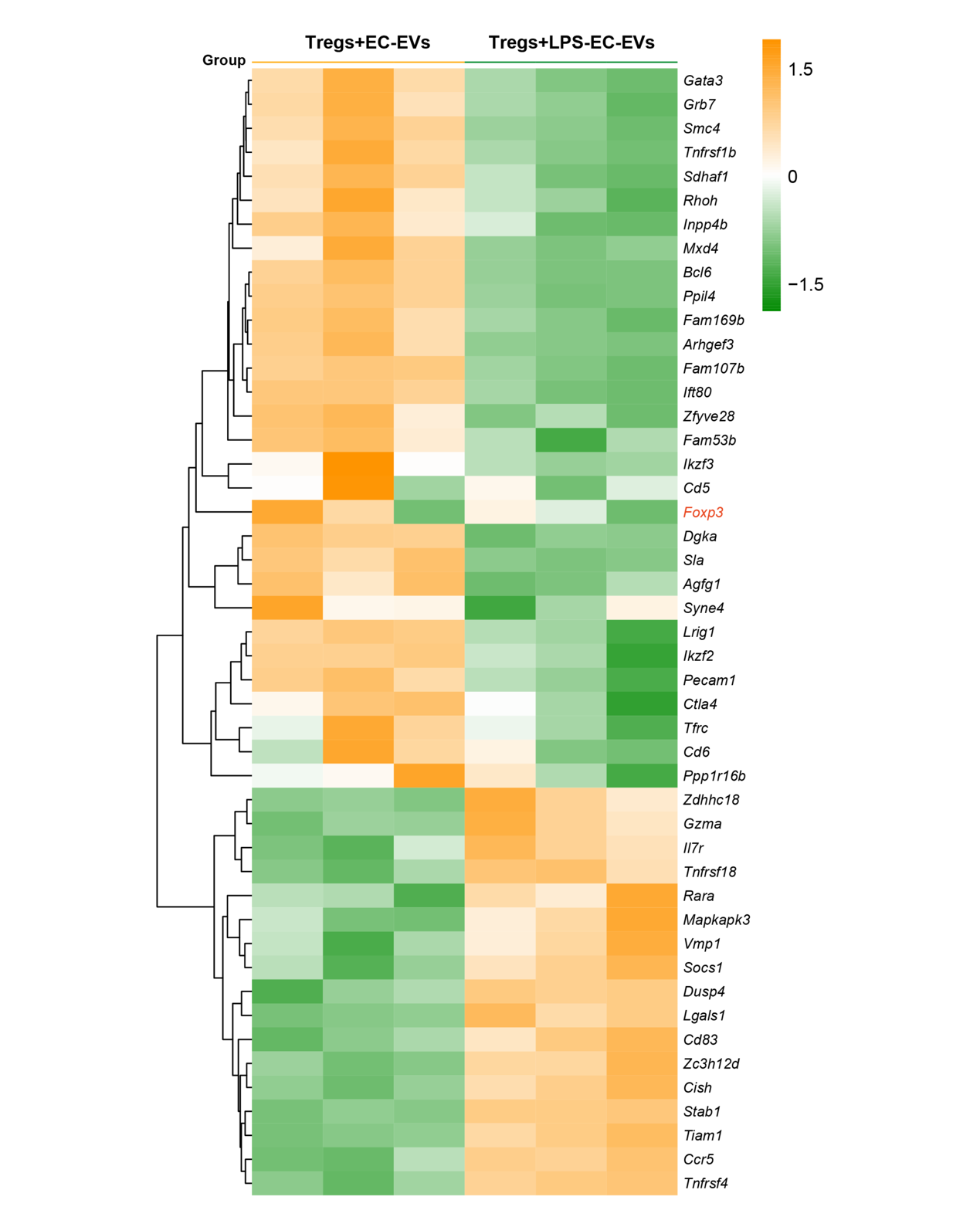


**FIGURE S13 Transcriptome analysis of Treg signature genes.**

**
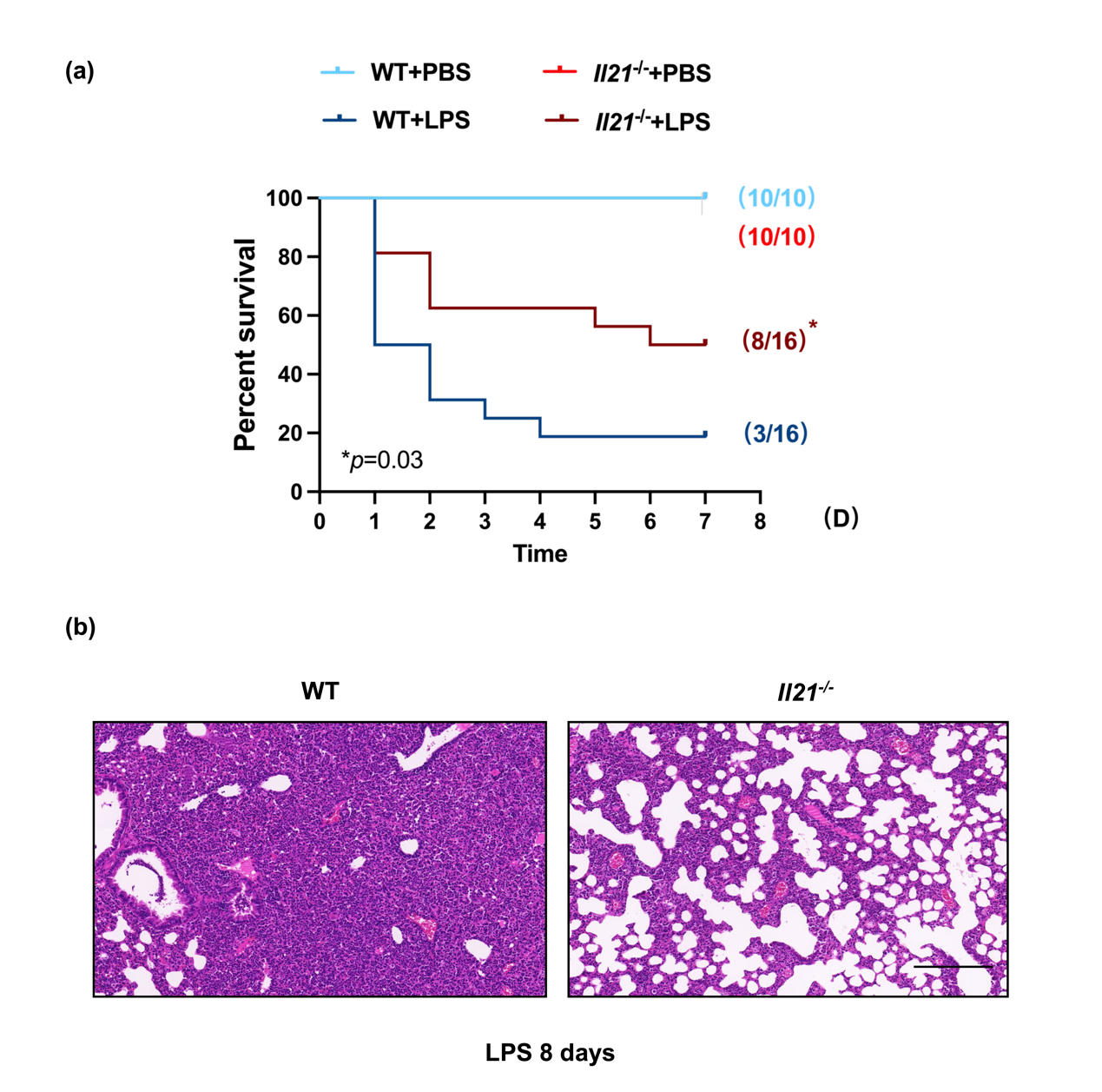
**

**FIGURE S14 Comparison of the survival rates after severe ALI between the** ***Il21^-/-^* and** **WT mice groups.** (a) The survival rates after severe ALI in the *Il21^-/-^* and WT mice. (b) Representative HE staining of lung tissues from survived *Il21^-/-^* and WT mice. Scale bar, 100 μm.


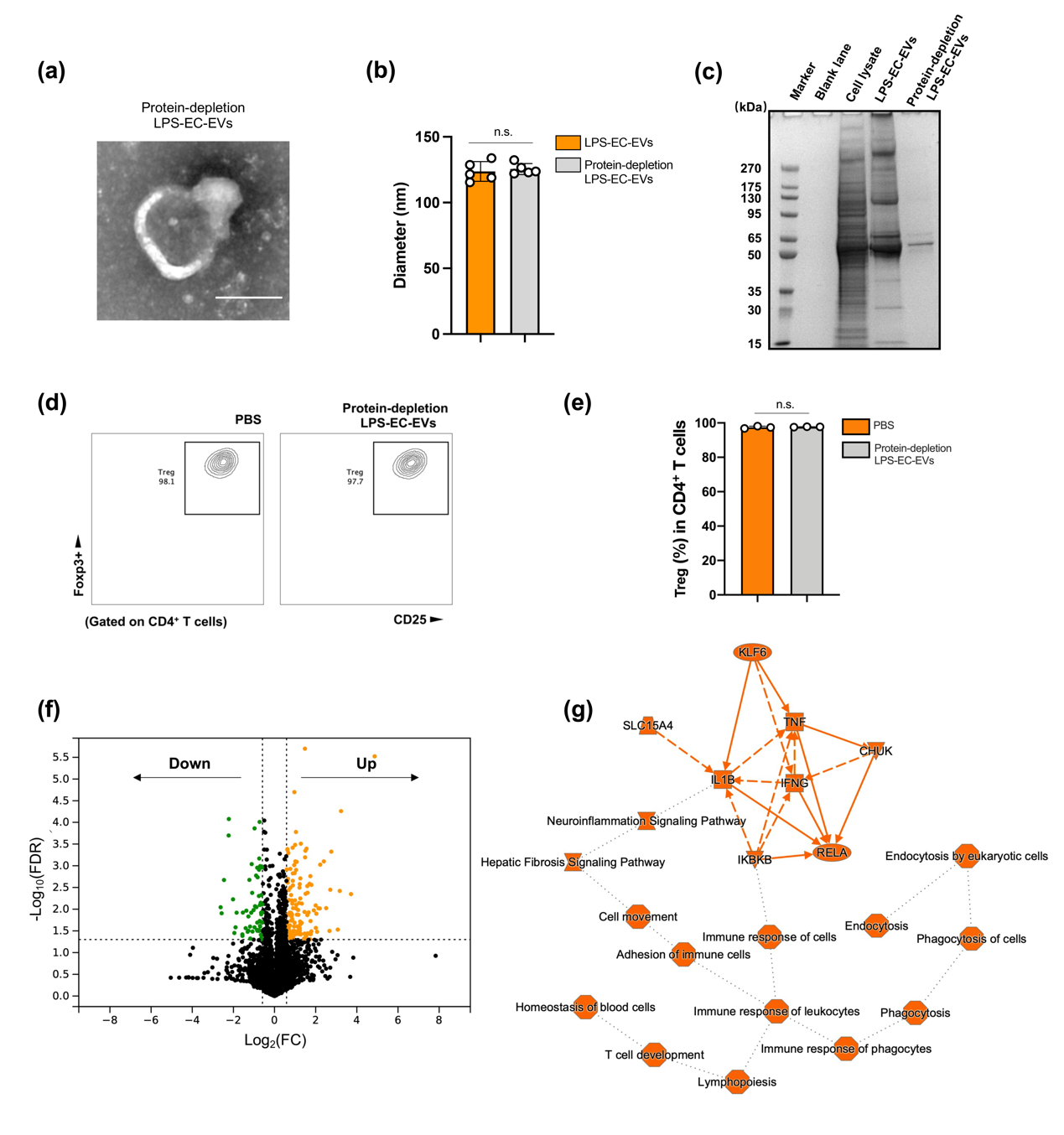


**FIGURE S15 Proteins in LPS-EC-EVs as functional cargoes to restrict Treg induction.** (a) Typical TEM image of protein-depletion LPS-EC-EVs. Scale bar, 100 nm. (b) Diameters of protein-depleted LPS-EC-EVs (n=5). (c) Coomassie blue staining indicated that the proteins in LPS-EC-EVs were almost depleted after Triton-100 and Proteinase K treatment. (d-e) FCM (d) and quantification (e) of Tregs exposed to either PBS or protein-depleted LPS-EC-EVs (n=3). (f) Volcano plot of the proteins that increased (orange, fold change>1.5, *p* value<0.05) and decreased (green, fold change<0.6, *p* value<0.05) in the LPS-EC-EVs, in comparison with those in the EC-EVs (n=3 / per group). (g) Graphic overview of the upregulated proteins in LPS-EC-EVs generated by IPA.


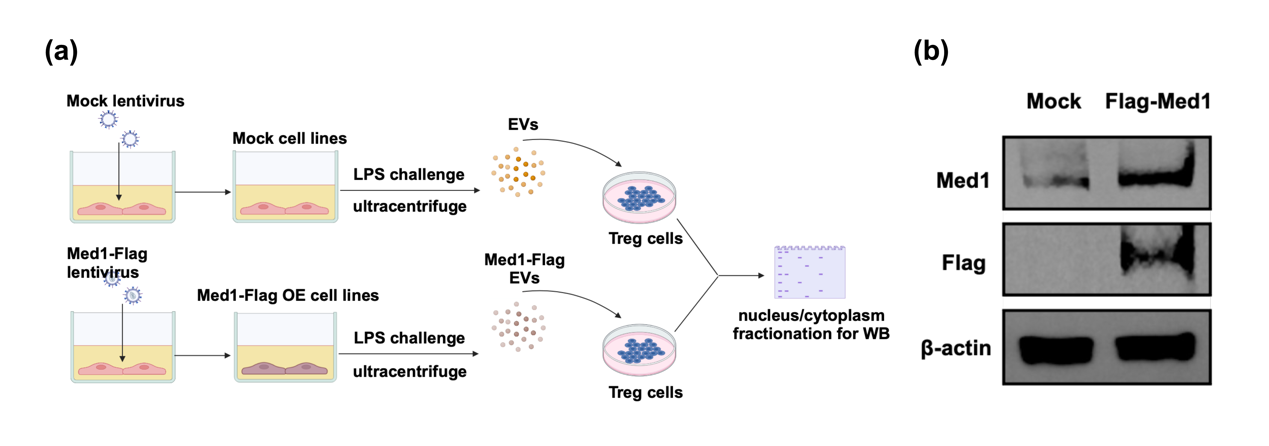


**FIGURE S16 Establishment of Flag-Med1 expression in MPMECs.** (a) Schematic diagram. (b) Immunoblotting analysis of Flag and Med1 expression in MPMECs after lentiviral transduction.


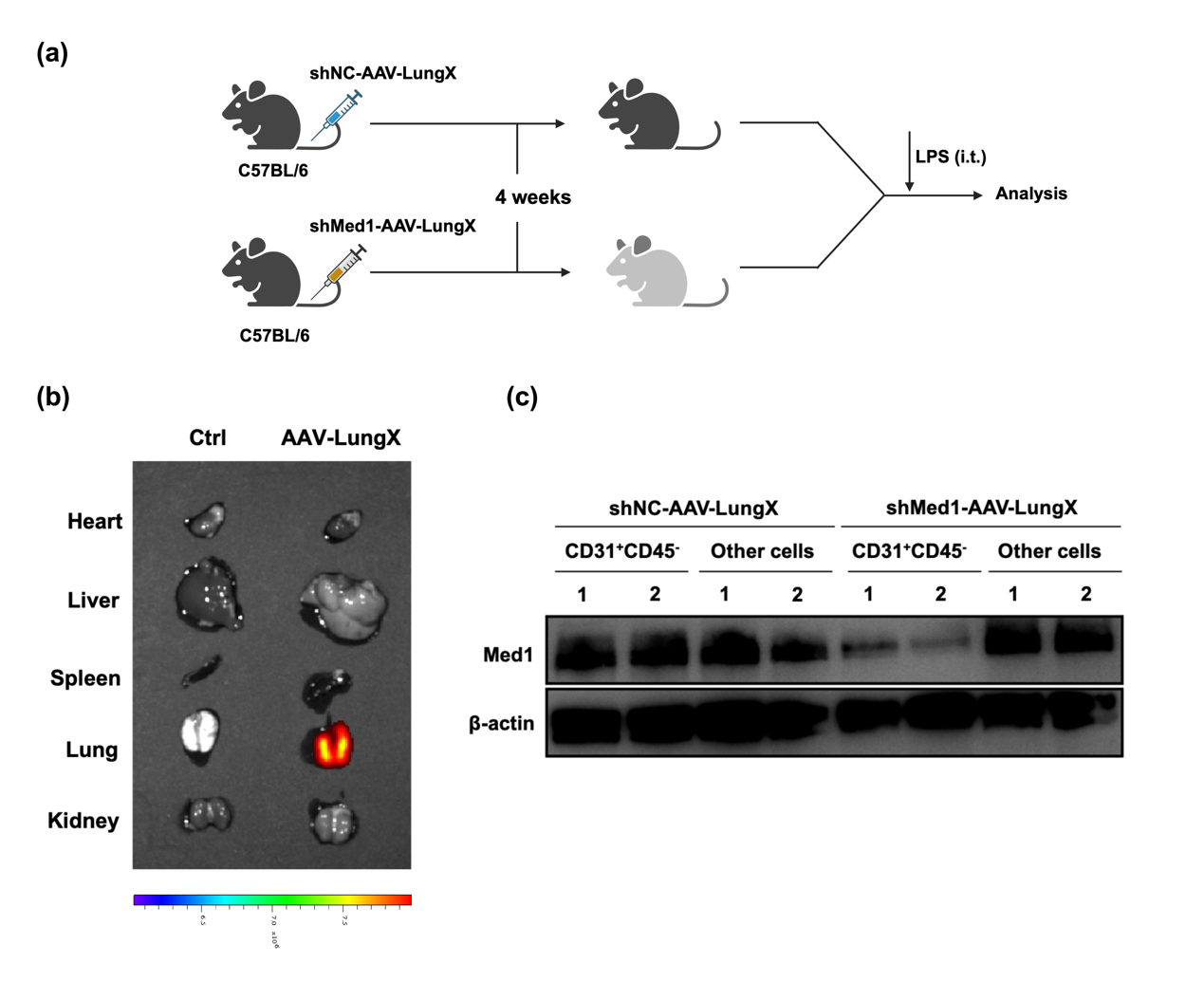


**FIGURE S17 AAV-mediated *in vivo* Med1 KD in pulmonary ECs.** (a) Schematic diagram (created using Biorender.com). (b) Representative fluorescence image of organs four weeks post AAV injection. (c) Immunoblotting analysis of Med1 expression in isolated pulmonary ECs from shMed1-AAV-LungX group mice.


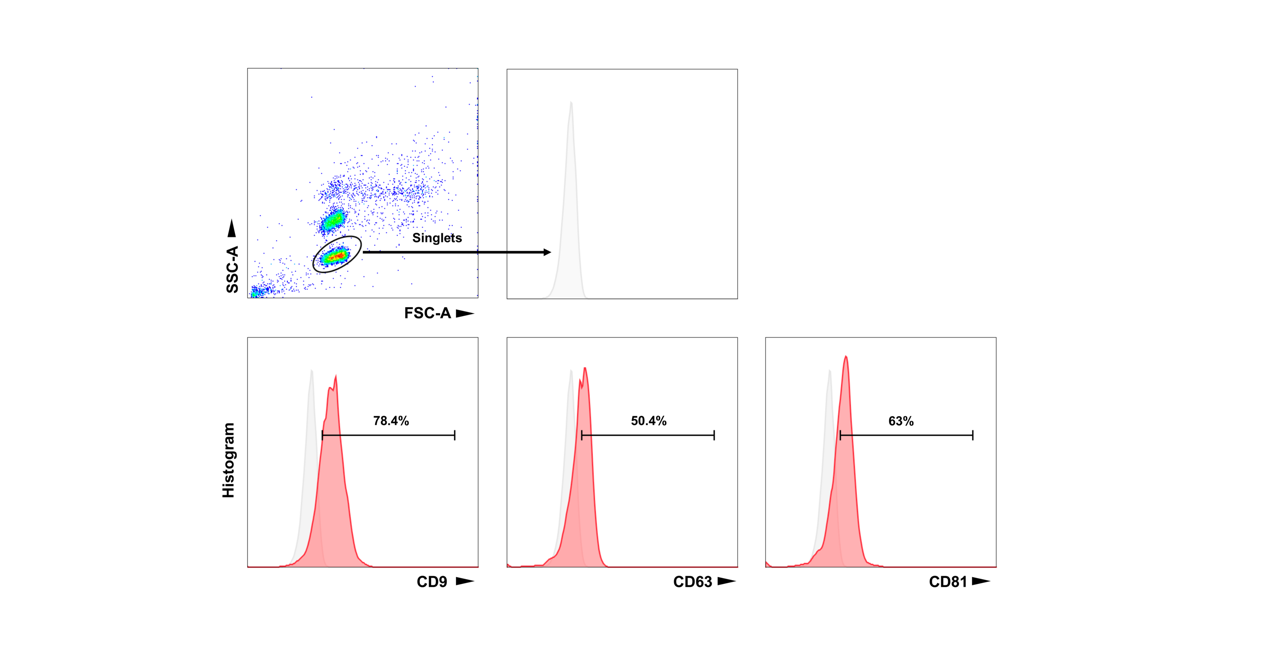


**FIGURE S18 Magnetic beads-based FCM analysis of BALF-EVs from patients.**
